# Viral DNA Sensors IFI16 and Cyclic GMP-AMP Synthase Possess Distinct Functions in Regulating Viral Gene Expression, Immune Defenses, and Apoptotic Responses during Herpesvirus Infection

**DOI:** 10.1128/mBio.01553-16

**Published:** 2016-11-15

**Authors:** Benjamin A. Diner, Krystal K. Lum, Jared E. Toettcher, Ileana M. Cristea

**Affiliations:** Department of Molecular Biology, Princeton University, Princeton, New Jersey, USA

## Abstract

The human interferon-inducible protein IFI16 is an important antiviral factor that binds nuclear viral DNA and promotes antiviral responses. Here, we define IFI16 dynamics in space and time and its distinct functions from the DNA sensor cyclic dinucleotide GMP-AMP synthase (cGAS). Live-cell imaging reveals a multiphasic IFI16 redistribution, first to viral entry sites at the nuclear periphery and then to nucleoplasmic puncta upon herpes simplex virus 1 (HSV-1) and human cytomegalovirus (HCMV) infections. Optogenetics and live-cell microscopy establish the IFI16 pyrin domain as required for nuclear periphery localization and oligomerization. Furthermore, using proteomics, we define the signature protein interactions of the IFI16 pyrin and HIN200 domains and demonstrate the necessity of pyrin for IFI16 interactions with antiviral proteins PML and cGAS. We probe signaling pathways engaged by IFI16, cGAS, and PML using clustered regularly interspaced short palindromic repeat (CRISPR)/Cas9-mediated knockouts in primary fibroblasts. While IFI16 induces cytokines, only cGAS activates STING/TBK-1/IRF3 and apoptotic responses upon HSV-1 and HCMV infections. cGAS-dependent apoptosis upon DNA stimulation requires both the enzymatic production of cyclic dinucleotides and STING. We show that IFI16, not cGAS or PML, represses HSV-1 gene expression, reducing virus titers. This indicates that regulation of viral gene expression may function as a greater barrier to viral replication than the induction of antiviral cytokines. Altogether, our findings establish coordinated and distinct antiviral functions for IFI16 and cGAS against herpesviruses.

## IMPORTANCE

How mammalian cells detect and respond to DNA viruses that replicate in the nucleus is poorly understood. Here, we decipher the distinct functions of two viral DNA sensors, IFI16 and cGAS, during active immune signaling upon infection with two herpesviruses, herpes simplex virus 1 (HSV-1) and human cytomegalovirus (HCMV). We show that IFI16 rapidly oligomerizes at incoming herpesvirus genomes at the nuclear periphery to transcriptionally repress viral gene expression and limit viral replicative capacity. We further demonstrate that IFI16 does not initiate upstream activation of the canonical STING/TBK-1/IRF3 signaling pathway but is required for downstream antiviral cytokine expression. In contrast, we find that, upon DNA sensing during herpesvirus infection, cGAS triggers apoptosis in a STING-dependent manner. Our live-cell imaging, mass spectrometry-based proteomics, CRISPR-based cellular assays, and optogenetics underscore the value of integrative approaches to uncover complex cellular responses against pathogens.

## INTRODUCTION

In mammalian cells, recognition of viral DNA is essential for the onset of antiviral responses during infection. This recognition is accomplished by constitutively expressed “DNA sensor” proteins, which bind foreign DNA and elicit the secretion of cytokines, such as type I interferons (IFN). While sensing was initially thought to occur only in subcellular compartments devoid of cellular DNA, we and others have recently demonstrated that interferon-inducible protein 16 (IFI16) binds to DNA of nucleus-replicating herpesviruses and stimulates cytokine expression within infected nuclei (1–4). The mechanisms by which IFI16 coordinates downstream signaling components from within the nucleus remains unclear. Currently, it is well established that, upon DNA stimulation, signaling is propagated in the cytosol first through stimulator of interferon genes (STING) (5, 6), then serine/threonine protein kinase TANK-binding kinase 1 (TBK-1) (7) and interferon response factor 3 (IRF3) (8, 9). IRF3 dimerizes and translocates to the nucleus, where it engages transcriptional regulators to induce cytokine expression (8–11). Ablation of these signaling components severely attenuates antiviral responses to foreign DNA and DNA viruses both in tissue culture and *in vivo* (6, 12–14). To date, several DNA sensors that activate the canonical STING/TBK-1/IRF3 signaling axis have been proposed, including IFI16 (4–6) and, most recently, the DNA sensor cyclic GMP-AMP synthase (cGAS) (15–18).

IFI16 was also proposed to stimulate other cellular pathways upon its binding to viral DNA. Several reports assert that DNA of herpesviruses Kaposi’s sarcoma-associated herpesvirus (KSHV), Epstein-Barr virus (EBV), and herpes simplex virus 1 (HSV-1) during infection assembles an IFI16-containing oligomeric structure termed the “inflammasome” (1, 19, 20). IFI16 was also reported to sense HIV-1 proviral DNA in nuclei of infected CD4^+^ T lymphocytes, leading to programmed cell death (21, 22). Finally, we and others have shown that IFI16 associates with subnuclear ND10 bodies and is targeted for degradation during HSV-1 infection (3, 23–25). ND10 bodies are known to associate with deposited herpesviral genomes and transcriptionally regulate viral and cellular genes (26). The role of IFI16 in regulating cellular and viral transcriptional activities has also been investigated (5, 27, 28). However, the molecular mechanisms regulating these dynamic IFI16 behaviors during herpesvirus infection remain largely unknown.

Here, we used a hybrid approach to decipher both distinct and cooperative functions of the DNA sensors IFI16 and cGAS in mediating antiviral responses to herpesviruses. Live-cell imaging, optogenetics, and proteomics were used to define IFI16 functions in space and time following infections with HSV-1 and human cytomegalovirus (HCMV). The distinct localizations, interactions, and antiviral functions were assigned to individual domains of IFI16 (pyrin or HIN). Using clustered regularly interspaced short palindromic repeat (CRISPR)/Cas9-based genetic tools in primary human cells, we delineated IFI16- and cGAS-mediated signaling pathways within the context of these dynamic behaviors.

## RESULTS

### IFI16 undergoes multiphasic subnuclear dynamics during HSV-1 and HCMV infections.

**FIG 1:**
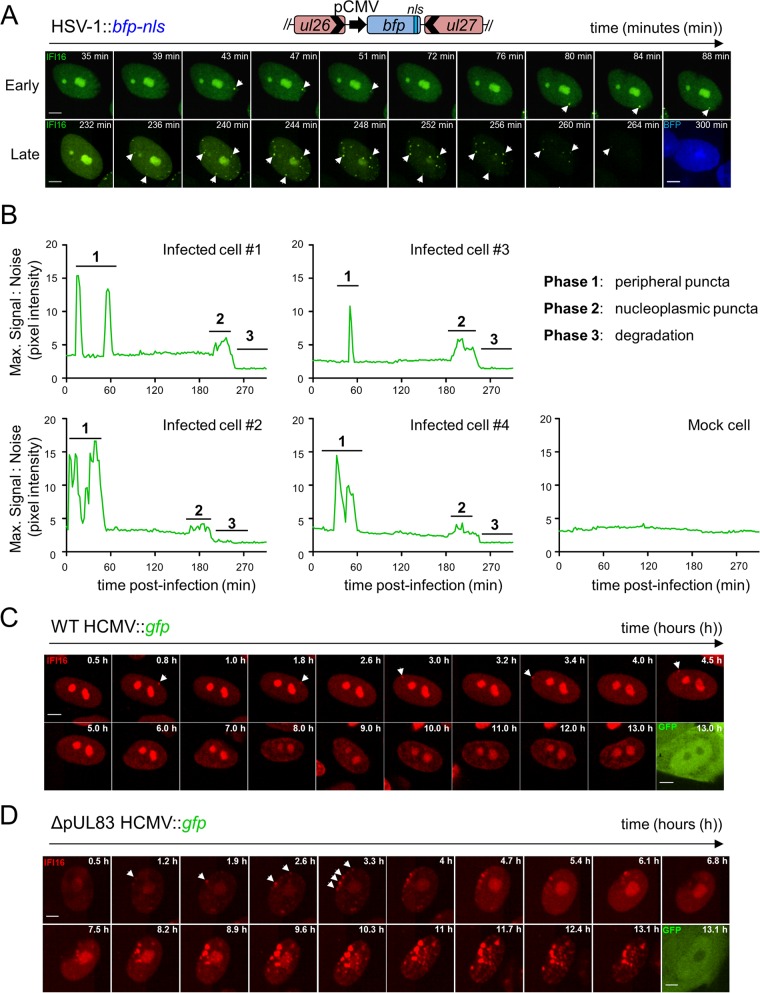
Multiphasic redistribution of IFI16 during both HSV-1 and HCMV infections. (A) Schematic of IFI16-eGFP fusion and HSV-1::*bfp-nls* recombinant virus containing a pCMV-*bfp-nls* expression cassette (top). HFFs expressing IFI16-eGFP were infected with HSV-1::*bfp-nls* (MOI of 10), and IFI16 was monitored by live-cell confocal fluorescence microscopy. Dynamic puncta are indicated (white arrowheads). Bars, 5 µm. (B) Maximum signal/noise pixel intensity ratios calculated in ImageJ are plotted as a function of time (in minutes). The characteristic phases of IFI16 are labeled and defined. (C and D) HFFs expressing IFI16-FusionRed were infected with either WT HCMV::*gfp* (MOI of 3) (C) or ΔpUL83 HCMV::*gfp* (MOI of 3) (D), and IFI16 was monitored and annotated as described above for panel A. See also Movie S1 and Fig. S1 in the supplemental material.

We and others have shown that IFI16 is recruited to subnuclear foci and is degraded during HSV-1 infection (3, 23, 24). However, in order to understand the underlying mechanisms and functions of IFI16, a finer spatial and temporal resolution of this dynamic behavior is necessary. We, therefore, developed a live-cell imaging platform for tracking IFI16 in real time. To mark infected nuclei, we generated an HSV-1 virus expressing a blue fluorescent tag (HSV-1::*bfp-nls*). Primary human foreskin fibroblasts (HFFs) stably expressing enhanced green fluorescent protein-tagged IFI16 (IFI16-eGFP) were infected with HSV-1::*bfp-nls* and imaged by live-cell microscopy (Fig. 1A). We observed rapid IFI16-eGFP puncta formation within the first 2 h postinfection (hpi). These puncta both assembled and disassembled on the order of minutes (Fig. 1B) and were exclusively formed at the nuclear periphery. After this initial phase, IFI16 localization transiently returned to that of mock-infected cells, i.e., nucleolar and diffuse nucleoplasmic localization (see Fig. S1B in the supplemental material). After several additional hours of infection, IFI16-eGFP appeared eliminated from nucleoli and redistributed into puncta dispersed throughout the nucleoplasm (Fig. 1A). Once maximal puncta formation was achieved, the IFI16-EGFP signal intensity diminished throughout the nucleus. While the number of peripheral puncta varied among infected cells, the temporality of IFI16 dynamics was consistent (Fig. 1B).

To test whether these IFI16 dynamics are common across herpesviruses, we monitored IFI16 behaviors during HCMV::*gfp* infection. Similar to HSV-1 infection, IFI16 assembled at nuclear peripheral foci early in infection (Fig. 1C). However, neither the second phase of nucleoplasmic puncta nor the degradation phase was observed for wild-type (WT) HCMV infection. We previously reported that the HCMV major tegument protein pUL83 inhibits IFI16 by blocking oligomerization via its pyrin domain (PY) (4). We, therefore, infected HFFs with an HCMV mutant lacking pUL83 expression (ΔpUL83::*gfp*). IFI16 maintained its redistribution into nuclear peripheral foci early in infection; however, these foci grew in both number and size as infection progressed. Initiation of early puncta formation displayed similar kinetics during HSV-1 and HCMV infection.

**FIG 2:**
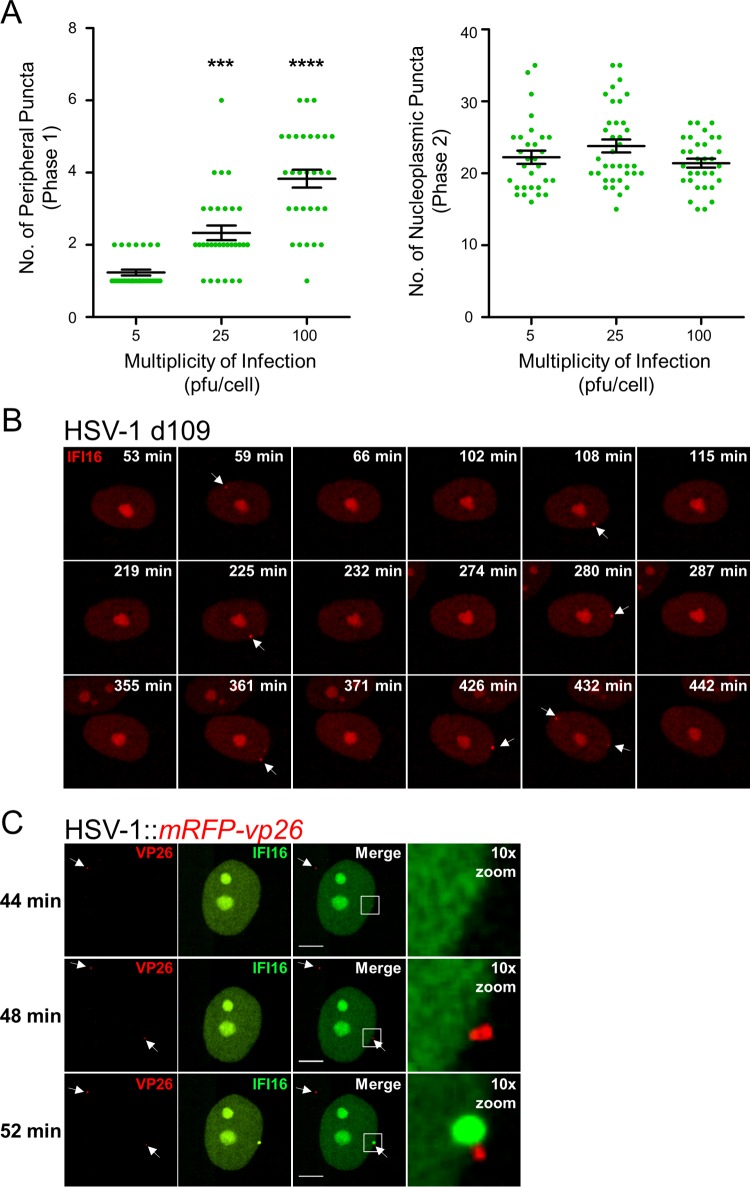
IFI16 localizes to sites of HSV-1 DNA deposition in an MOI-dependent manner. (A) Number of IFI16-eGFP peripheral (phase 1, left panel) and nucleoplasmic puncta (phase 2, right panel) observed in HSV-1::*bfp-nls-*infected HFFs at various viral loads (plaque-forming units/cell). Each green dot symbol represents the number of puncta observed per cell. Values are means (black line) ± standard errors of the means (SEMs) (*n* = 30) (error bars). Values that are significantly different by one-way ANOVA are indicated by asterisks as follows: ***, *P* < 0.001; ****, *P* < 0.0001. (B) HFFs expressing IFI16-FusionRed were infected with HSV-1 *d109* infection (MOI of 10) and monitored by live-cell fluorescence confocal microscopy. Dynamic puncta are indicated (white arrowheads). Images are at the same magnification as shown in panel C. (C) As in panel B, IFI16-eGFP during HSV-1::*mRFP-vp26* infection (MOI of 10). IFI16 was monitored and annotated as described above for panel B. Red fluorescent HSV-1 capsids are indicated (white arrowheads). Bar, 5 µm. See also Movie S2 in the supplemental material.

The localization of IFI16 to nuclear peripheral foci upon herpesvirus infections and its well-characterized binding to viral DNA (6) suggest that these foci may be sites of viral DNA deposition. Consistent with this hypothesis, when we monitored the localization of endogenous IFI16 in newly infected cells at the edge of a developing plaque, we observed the formation of asymmetric puncta at the nuclear periphery (see Fig. S1A in the supplemental material). Additionally, we assessed the dependence of puncta formation on the multiplicity of infection (MOI). We observed that increasing the number of infectious viral particles per cell increased the number of transient IFI16 foci at the nuclear periphery (phase 1) (Fig. 2A, left), but not the number of nucleoplasmic foci formed just prior to IFI16 degradation (phase 2) (Fig. 2A, right). To further explore this, we monitored IFI16 localization upon infection with several recombinant HSV-1 strains. The HSV-1 *d106*::*gfp* virus harbors deletions in four of five HSV-1 immediate early (IE) transactivation genes (*icp4*, *icp22*, *icp27*, and *icp47*), expressing only the IE gene *icp0* that encodes the viral E3 ubiquitin ligase ICP0, previously shown as required for IFI16 degradation (24). The three phases observed during HSV-1::*bfp-nls* infection were conserved during HSV-1 *d106*::*gfp* infection (Fig. S2). This suggests that the combination of HSV-1 DNA and ICP0 activity is sufficient for eliciting all three characteristic IFI16 phases. In contrast, phases 2 and 3 were not observed during infection with the HSV-1 *d109* mutant (Fig. 2B), which lacks all IE genes, rendering the HSV-1 genome transcription and replication incompetent. However, assembly and disassembly of nuclear peripheral IFI16 puncta (phase 1) were observed during *d109* infection, not only within the first hours but also during later hours of infection. This substantiates that peripheral foci form at the location of HSV-1 genome deposition. We next infected IFI16-eGFP-expressing fibroblasts with HSV-1::*mrfp-vp26* virus, in which the major HSV-1 capsid protein VP26 is fused to monomeric red fluorescent protein (mRFP). Attachment of red HSV-1 capsids at the outer nuclear membrane instantaneously induced formation of IFI16 aggregates at adjacent sites in the nucleus (Fig. 2C). Diffusing capsids not present at the nuclear periphery did not appear to influence IFI16 localization. Altogether, these data indicate that IFI16 aggregates at the nuclear periphery in response to the incoming herpesviral DNA genome.

### The pyrin domain mediates IFI16 nuclear peripheral recruitment and degradation, while the HIN200 domains mediate redistribution to centromeres during HSV-1 infection.

**FIG 3:**
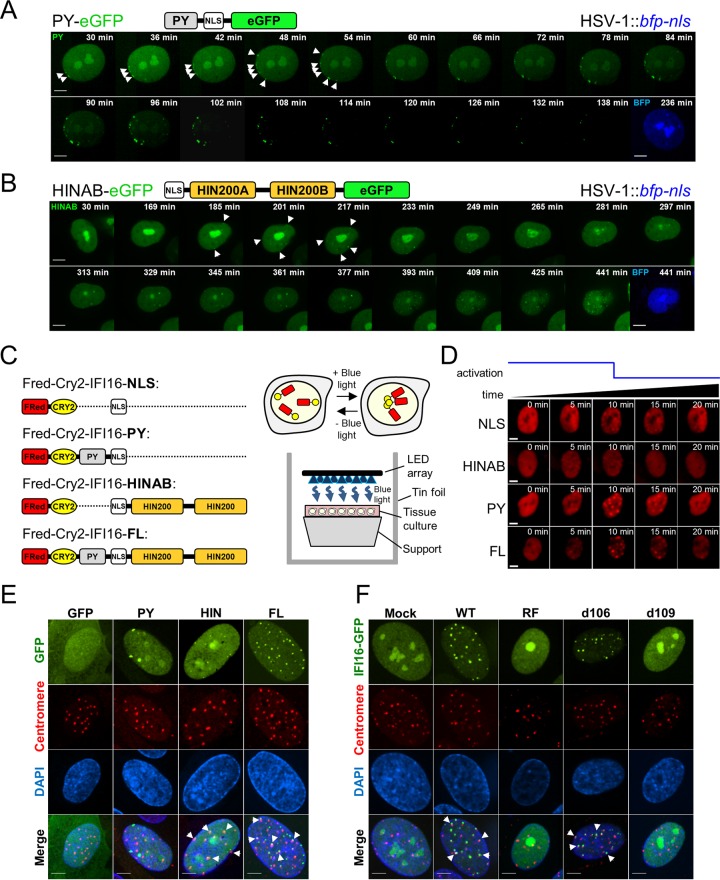
IFI16 PY and HIN domains display distinct behaviors during HSV-1 infection. (A and B) HFFs expressing either IFI16-PY-eGFP (A) or IFI16-HINAB-eGFP (b) infected with HSV-1::*bfp-nls* (MOI of 10) and imaged live by confocal fluorescence microscopy. Schematic representations of eGFP-tagged IFI16 derivatives are displayed above the images; pyrin (PY), HINAB (two HIN200 domains), and nuclear localization signal (NLS) are shown. Dynamic puncta are indicated (white arrowheads). Bars, 5 µm. (C, left) Schematic of FusionRed-CRY2^olig^-IFI16 fusions. FRed, FusionRed. (Right) Concept of blue light-induced CRY2^olig^-mediated aggregation and experimental scheme. LED, light-emitting diode. (D) FusionRed-CRY2^olig^-IFI16 fusions expressed in HFFs were induced to assemble (min 0 to 10) and disassemble (min 10 to 20). “Activation” represents application of blue light. Bars, 5 µm. (E) HFFs expressing IFI16 domain-eGFP fusions or eGFP alone infected with WT HSV-1 (MOI of 10) are stained for centromeres (anticentromere antibodies) at 3 hpi. Overlap between eGFP constructs (green) and centromeres (red) are shown (white arrowheads). DAPI, 4′,6′-diamidino-2-phenylindole. Bars, 5 µm. (F) As in panel E, WT, RF, *d106*, and *d109* infection (MOI of 10). See also Movie S3 in the supplemental material.

To determine which IFI16 domains mediate its distinct virus-induced behaviors, we generated HFFs expressing either the N-terminal PY (PY-eGFP) or two HIN200 (HINAB-eGFP) domains of IFI16 (Fig. 3A and B). Both constructs retained the nuclear localization signal (NLS) and displayed characteristic IFI16 nucleoplasmic and nucleolar distribution in uninfected cells. However, upon HSV-1::*tagBFP2* infection, only the PY-eGFP fusion was recruited to the nuclear periphery early in infection (Fig. 3A). As observed with the ΔpUL83 HCMV::*gfp* virus (Fig. 1D), additional foci appeared directly adjacent to the earlier ones, asymmetrically propagating along the edge of the nucleus. We further confirmed that the recruitment of PY to the nuclear periphery is not likely derived from interactions with endogenous IFI16 by demonstrating a similar PY localization in infected HEK293 cells, which do not seem to express IFI16 (see Fig. S3A in the supplemental material) (1–4). Moreover, we observed degradation of the PY-eGFP fusion as early as 1 hpi.

Several groups, including ours, have shown that an HSV-1 mutant lacking the E3 ubiquitin ligase activity of the IE viral protein ICP0 from mutations in its ring finger domain (RF) is attenuated in its ability to induce IFI16 degradation (3, 24). Thus, to confirm that the PY domain is targeted for degradation, HFFs expressing eGFP-tagged IFI16 domains or eGFP control were infected with either WT or RF HSV-1 virus. In agreement with our microscopy data, full-length (FL) IFI16- and PY-eGFP fusion proteins were degraded upon WT, but not RF HSV-1 infection (see Fig. S3B in the supplemental material). These results were recapitulated in infected Flp-In HEK293 cells inducibly expressing the same constructs (Fig. S3C and S3D).

The targeting of the PY domain for degradation by HSV-1, our live-cell imaging data, and our previous findings that HCMV pUL83 blocks PY oligomerization, suggest that the PY domain homotypic interactions are critical for IFI16 antiviral functions. We, therefore, developed optogenetic tools to achieve fine experimental control of IFI16 oligomerization state. The IFI16 domains were fused to the photoreceptor cryptochrome 2 (CRY2-IFI16), allowing for their reversible, blue light-induced clustering through homo-oligomerization of CRY2 (Fig. 3C). A FusionRed fluorescent protein further allowed imaging of activation and relaxation of IFI16 fusion protein clustering. We observed maximal clustering of all constructs within 10 min of initiating light stimulation and absolute system relaxation within 10 min of ceasing light stimulation (Fig. 3D). CRY2-IFI16 fusions lacking the PY domain clustered weakly, whereas both CRY2-PY and full-length CRY2 fusion proteins displayed prominent clustering. This indicates that the IFI16 PY domain alone can increase the efficiency of CRY2 oligomerization, underscoring its function in assembly of IFI16 foci during viral infection. Altogether, these data demonstrate that the PY domain is sufficient for IFI16 oligomerization and early aggregation at the nuclear periphery.

In contrast to the PY domain of IFI16, HINAB-eGFP fusions did not form peripheral puncta, nor were they degraded, in response to HSV-1. Rather, localization of the HINAB-eGFP fusion was static during early stages of infection, initially displaying no redistribution. Beginning at 3 to 6 hpi, HINAB-eGFP puncta slowly began to accumulate throughout the nucleoplasm (phase 2) (Fig. 3B). These foci slowly grew in frequency, remained stable over time, and were noticeably smaller than corresponding phase 2 puncta observed for full-length IFI16. Furthermore, the average number of these phase 2 puncta (approximately 23) did not vary with viral loads and matched the number of centromeres within human cell nuclei (Fig. 2A, left). Indeed, we observed colocalization of phase 2 IFI16 FL-eGFP and IFI16 HINAB-eGFP puncta with centromeres (Fig. 3E). Previous reports have suggested that proteasomal degradation of centromeric proteins by HSV-1 ICP0 E3 ubiquitin ligase activity induces a centromeric instability (29, 30). In agreement, we observed IFI16 foci overlap with centromere protein complexes and ICP0 during infection with HSV-1 viruses with functional ICP0 (WT and *d106*), but not those lacking either ICP0 ubiquitylation activity (RF) or ICP0 expression (*d109*) (Fig. 3F; see Fig. S3E in the supplemental material). Thus, IFI16 may be involved in a centromere destabilization pathway upon HSV-1 infection through the recruitment of its HIN200 domains to centromeres.

### The pyrin domain of IFI16 mediates the interaction with ND10 bodies and cGAS.

**FIG 4:**
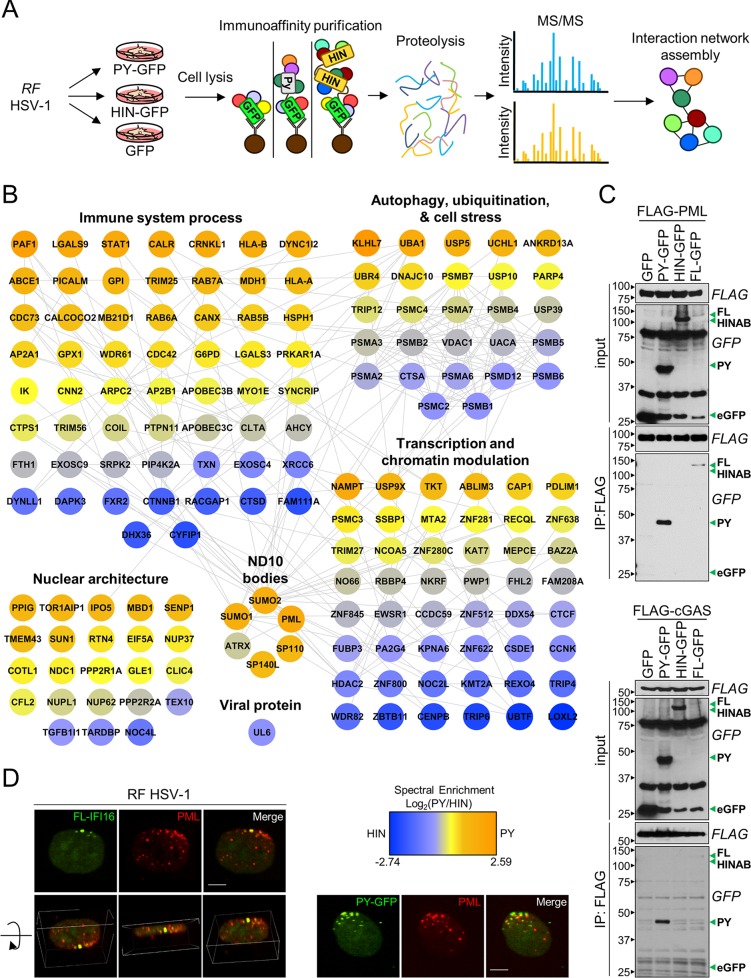
IFI16 interacts with PML and cGAS through the PY domain. (A) Scheme for immunoaffinity purification-mass spectrometry-based identification of IFI16 domain protein interactions in RF HSV-1-infected HFFs (MOI of 10) at 6 hpi. (B) Specificity (SAINT)-filtered IFI16-PY and IFI16-HIN interaction networks during RF HSV-1 were generated with the STRING database and rendered with Cytoscape. Colors represent relative spectral abundances of interactions enriched in either PY (orange) or HIN (blue) domain isolations. (C) Western blots of reciprocal immunoaffinity isolations in HEK293T cells cotransfected with the indicated IFI16-eGFP fusions (green arrowheads) and either FLAG-PML or FLAG-cGAS. The positions of molecular mass markers (in kDa) are shown to the left of the blots. (D) Immunofluorescence microscopy of indicated IFI16-eGFP fusions and PML in HFFs. A 3D image was rendered from Z-stacks. Bars, 5 µm. See also Fig. S2 in the supplemental material.

The dynamic localizations of the PY and HIN domains during HSV-1 infection prompted us to explore the factors mediating these distinct behaviors. Therefore, we investigated IFI16 domain-specific protein interactions in RF HSV-1-infected fibroblasts. PY and HINAB interactions were determined by immunoaffinity purification (IP) and tandem mass spectrometry (MS/MS) (Fig. 4A). Following SAINT specificity filtering, interaction networks were assembled (Fig. 4B; see Table S1 in the supplemental material). In agreement with the known housekeeping functions of IFI16, the resulting functional interaction network contained proteins involved in innate immunity, cell stress, and transcription regulation.

Consistent with its DNA binding activities, relative to the PY domain, the HIN domains were enriched up to 7-fold in proteins involved in modulating epigenetics and transcription, including LOXL2, WDR82, and KMT2A. Furthermore, chromatin-associated proteins, such as FAM111A, a known restriction factor of viral genome replication, were additionally enriched toward the HIN domain. We also found a strong HIN association with the centromeric DNA-binding protein CENP-B (centromere protein B). Of note, the viral portal protein UL6 was found enhanced in association with the HIN domains, consistent with a role for these domains in binding the naked viral DNA genome upon its deposition into the nucleus.

PY domain interactions were enriched in nuclear architecture and nuclear pore complex proteins, such as TOR1AIP1, SUN1, RTN4, TMEM43, and NUP37. This agrees with the localization of the PY domain to the nuclear periphery early in infection (Fig. 3A). Other PY-enriched proteins have known roles in ubiquitination and the proteasome degradation pathway, including KLHL7, UBA1, UCHL1, ANKRD13A, UBR4, and USP10. These interactions may be involved in the targeted degradation of PY during HSV-1 infection.

Of relevance to cellular immunity, both the antiviral ND10 body complex and the DNA sensor cGAS were PY-enriched associations during infection. ND10 body components included PML (promyelocytic leukemia protein), SUMO1, SUMO2, ATRX, SP110, and SP140L. To confirm these interactions, we performed reciprocal affinity isolation of FLAG-tagged PML and cGAS constructs with IFI16-eGFP fusions (Fig. 4C). Only IFI16-FL and the PY domain and were detected in PML and cGAS immunoisolates. The reciprocal results were obtained in isolations of the IFI16-eGFP domains (see Fig. S4 in the supplemental material). Furthermore, PML and cGAS were both identified as specific interactions with full-length IFI16 by IP-MS (Table S1).

We next examined PML and IFI16-PY organization during early RF HSV-1 infection. We observed similar asymmetric distribution of PY and PML at the nuclear periphery (Fig. 4D). Interestingly, the IFI16- and PML-containing structures do not appear to perfectly overlap. Nevertheless, our observations substantiate that the early recruitment of IFI16 to viral entry sites at the nuclear periphery is mediated by PY interactions with nuclear architecture proteins and triggers further recruitment of ND10 components.

### IFI16 is required for antiviral cytokine expression, but not for upstream activation of STING/TBK-1/IRF3 signaling.

**FIG 5:**
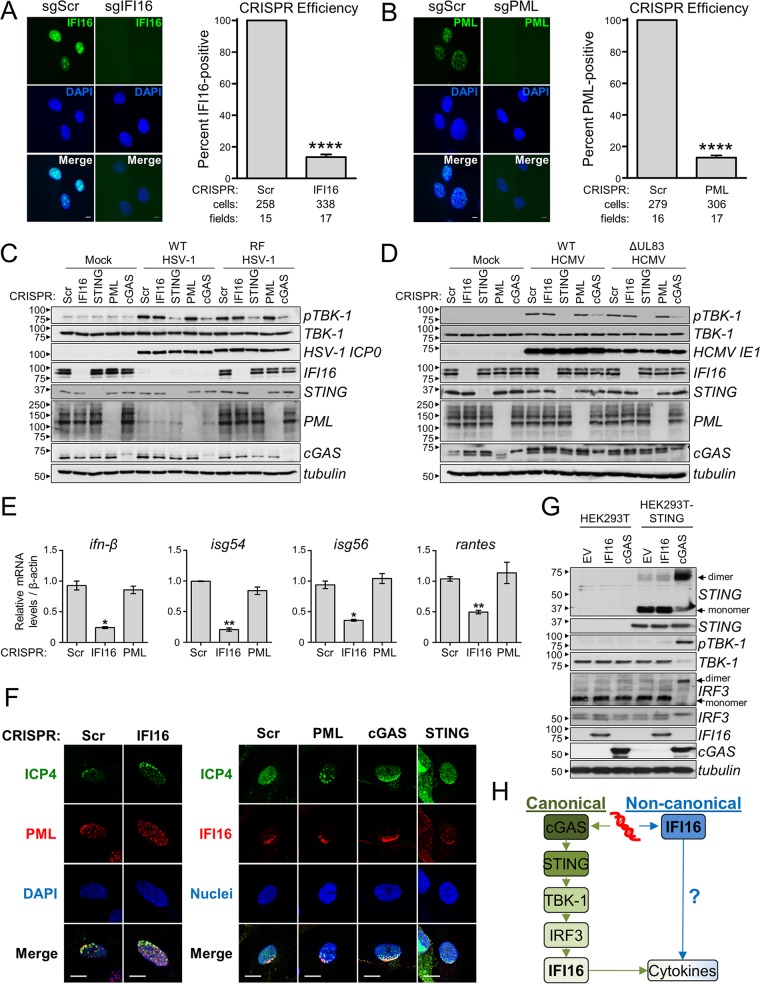
IFI16 is required for antiviral cytokine expression, but not activation of STING/TBK-1/IRF3 signaling. (A and B) CRISPR-mediated knockout of IFI16 (sgIFI16) (A) or PML (sgPML) (B) compared to scrambled sgRNA control cells (sgScr). (Left) CRISPR-HFFs were imaged by immunofluorescence microscopy. Bar, 5 µm. (Right) The average percentage of IFI16- or PML-positive cells per field (plus SEM) is plotted. Values that are significantly different (*P* ≤ 0.0001) from the value for the scrambled control by Student’s *t* test are indicated (****). The numbers of scored cells and fields are shown below the bars. (C) Western blots of CRISPR-HFFs infected with WT or RF HSV-1 (MOI of 10) at 6 hpi. (D) As in panel C, WT or ΔpUL83 HCMV infection (MOI of 3) at 6 hpi. (E) Cytokine mRNA levels in CRISPR-HFFs infected with RF HSV-1 (MOI of 10) at 6 hpi. Data were normalized to *β-actin*. Values are means ± SEMs (*n* = 2). Values that are significantly different from the value for the scrambled control by Student’s *t* test are indicated by asterisks as follows: *, *P* ≤ 0.05; **, *P* ≤ 0.01. (F) Immunofluorescence microscopy of ICP4 and PML (left panel) and of ICP4 and IFI16 (right panel) in CRISPR-HFFs (sgIFI16 versus sgScr and sgPML, sgcGAS, and sgSTING versus sgScr, respectively) upon RF HSV-1 infection (MOI of 0.1) at 24 hpi. A representative cell shown is at the edge of plaque. Bars, 10 µm. (G) Western blots of HEK293T or HEK293T-STING cells transfected with the indicated constructs. STING and IRF3 dimerization was tested by nonreducing SDS-PAGE and native PAGE, respectively. EV, empty vector. (H) Two proposed signaling cascades initiated in response to pathogenic DNA (red) by either cGAS (green) or IFI16 (blue). Question marks denote unknown signaling components. See also Fig. S2 in the supplemental material.

IFI16 was shown to bind viral DNA and to induce antiviral cytokines, and our findings indicate a likely viral DNA sensing event at the nuclear periphery early in herpesvirus infection. Current models assert that, upon binding to viral DNA, IFI16, like other DNA sensors such as cGAS, signals through the STING/TBK-1/IRF3 axis. However, it is unclear how a nucleus-derived IFI16-dependent signal is propagated to this cytoplasmic DNA sensing hub. We, therefore, used CRISPR/Cas9 technology (31) to test the requirement of IFI16 for activation of the STING/TBK-1/IRF3 axis with respect to cGAS and STING. As our findings suggest coordination between IFI16 and ND10 bodies, we also probed for PML requirement in immune signaling. We designed three candidate single guide RNAs (sgRNAs) for CRISPR targeting of *ifi16*, *sting*, *pml*, and *cgas* in HFFs (CRISPR-HFFs), which were tested by Western blotting and/or immunofluorescence microscopy to select those providing the most effective knockouts (Fig. 5A and B; see Fig. S5 in the supplemental material)*.*

Having validated the knockout efficiencies in primary fibroblasts, we infected CRISPR-HFFs with WT or RF HSV-1 (MOI of 10) or ΔpUL83 HCMV (MOI of 3) to assess immune activation. By Western blotting, stimulation of TBK-1, indicated by its phosphorylation at serine 172 (pTBK-1), was observed in all virally infected scrambled CRISPR control cells with respect to uninfected (Mock) cells (Fig. 5C and D). Interestingly, CRISPR knockout of IFI16 and PML had no observable effect on TBK-1 activation, whereas both cGAS and STING knockout drastically attenuated it. Nevertheless, CRISPR knockout of IFI16 did significantly suppress the induction of antiviral cytokines upon RF HSV-1 infection, whereas PML knockout did not (Fig. 5E). Additionally, knockout of IFI16 impeded the recruitment of PML to viral genome entry points at the nuclear periphery, whereas knockout of either cGAS or STING did not (Fig. 5F, left panel; see Fig. S5E in the supplemental material). We further demonstrated that knockout of either PML, cGAS, or STING did not impair the enrichment of IFI16 at viral genome entry points (Fig. 5F, right panel). We observed no difference in the localization of cGAS in the knockout of IFI16, PML, or STING (Fig. S5F). These data suggest that IFI16, while necessary for PML localization to incoming viral DNA and for antiviral cytokine expression, may not be required for upstream activation of the canonical STING signaling pathway upon HSV-1 or HCMV infection in fibroblasts.

To verify this, we tested immune activation in a HEK293T system, as these cells lack expression of endogenous STING and other established DNA sensors. To reconstitute a DNA sensing axis, STING was first stably introduced by lentiviral transduction (HEK293T-STING cells). Activation of DNA-dependent immune signaling was then assessed by transient overexpression of IFI16 or cGAS, with plasmid consequently serving as the DNA substrate. Consistent with the above results, pTBK-1 was observed only in the presence of both cGAS and STING, but not for IFI16 and STING (Fig. 5G). Furthermore, when using nonreducing SDS-PAGE and native PAGE to examine dimerization of STING and IRF3, respectively, we observed their dimerization only in response to STING and cGAS coexpression (Fig. 5G). Altogether, these results show that, while cGAS is an upstream activator of the STING/TBK-1/IRF3, IFI16 is not. Of consideration, transfected DNA substrates localize predominantly to the cytosol, where cytoplasmic cGAS is available to bind. In contrast, interaction of nuclear IFI16 with transfected DNA is less likely. As we and others have reported IFI16-dependent induction of cytokines upon viral infection (2–4, 6, 24), it is also possible that IFI16 may, in fact, function downstream of this canonical pathway (Fig. 5H, green pathway). IFI16 may also function within its own signal transduction pathway, in which it is the initiating DNA sensor, either activating cytokines directly or signaling through currently undetermined components (Fig. 5H, blue pathway).

### cGAS-dependent DNA sensing initiates apoptosis.

**FIG 6:**
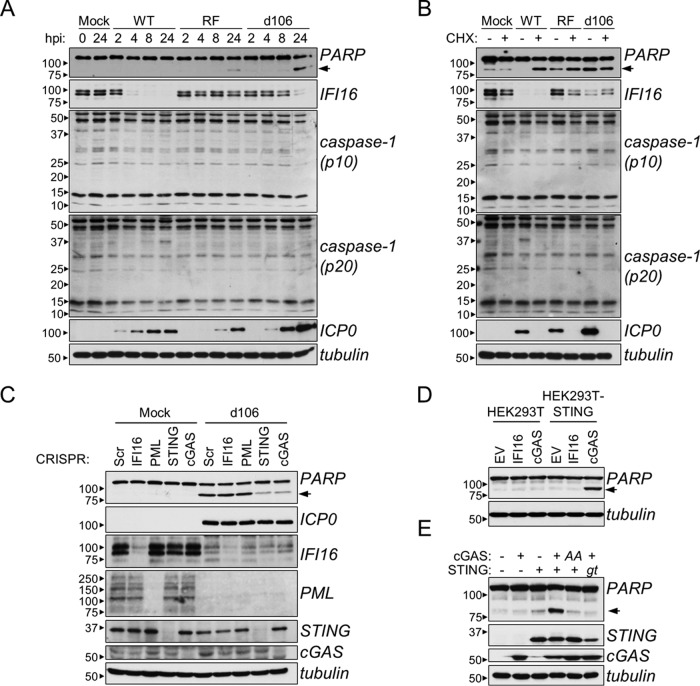
cGAS, not IFI16, is required for HSV-1 and DNA-dependent apoptosis. (A) Western blots of WT, RF, or *d106* HSV-1-infected HFFs (MOI of 10) at the indicated times. (B) Western blots of cycloheximide (CHX) (10 µg/ml) or mock-treated HFFs infected with HSV-1 as described above for panel A for 24 h. (C) Western blots of *d106* HSV-1-infected CRISPR-HFFs. (D) Western blot of HEK293T or HEK293T-STING cells transfected with indicated constructs for 16 h. (E) Western blots of HEK293T cells transfected as described above for panel D. *AA*, hcGAS GS212/213AA mutant; *gt*, human STING I200N mutation. For all panels, PARP cleavage is indicated by black arrowheads.

Although IFI16 does not initiate signaling through STING, it remains possible that its binding to viral DNA has additional antiviral consequences. For instance, IFI16 was reported to mediate CD4^+^ T-cell programmed cell death upon HIV-1 infection (21) and caspase-1-containing inflammasome formation upon herpesvirus infection (1, 19, 20). Moreover, the *d106* HSV-1 mutant was reported to initiate apoptotic cell death (32). Therefore, to further examine these IFI16 functions, HFFs were infected with WT, RF, and *d106* HSV-1. The activation of caspase-3 and inflammasome caspase-1 was assessed over 24 h by monitoring poly(ADP-ribose) polymerase (PARP) and caspase-1 cleavage, respectively. No change in caspase-1 cleavage was observed in any condition, suggesting the absence of inflammasome assembly (Fig. 6A). PARP cleavage was observed only at 24 hpi, being evident strongly in *d106* infection and weakly in RF infection. No PARP cleavage was observed in response to WT HSV-1-infected or mock-infected cells. These observations are consistent with others’ findings that a late-expressing viral factor blocks apoptotic cellular responses (33). Therefore, to block *de novo* viral protein synthesis, we treated HFFs with cycloheximide (CHX) following adsorption of WT, RF, and *d106* HSV-1 and assayed caspase activity after 24 h. With CHX treatment, all three viruses induced robust PARP cleavage relative to untreated or treated mock-infected cells (Fig. 6B). No caspase-1 activity was detected under any tested condition. Altogether, these results confirm that apoptosis is triggered upon HSV-1 infection and that a *de novo*-synthesized viral gene product can antagonize this cellular response. Furthermore, these data recapitulate previous findings that HSV-1 relies in part on E3 ubiquitin ligase activity of ICP0 (lost in the RF virus) for IFI16 degradation. However, IFI16 levels did not correlate with PARP cleavage levels, making it difficult to conclude its involvement in apoptosis.

To further test its role, we infected CRISPR-HFFs with the *d106* virus and assayed for caspase-3 activation. Unexpectedly, knockout of IFI16 and PML had no effect on PARP cleavage, while knockout of cGAS and STING strongly attenuated it (Fig. 6C). This indicates that viral DNA-induced signaling through the STING/TBK-1/IRF3 axis is an initiator of apoptotic responses. We further validated this result in the HEK293T system. Transient, plasmid-based coexpression of cGAS, but not IFI16, with stably expressed STING induced PARP cleavage (Fig. 6D). PARP cleavage responses were lost when WT cGAS was replaced with the GS212/213AA mutant that is unable to generate cyclic dinucleotides upon DNA stimulation (16, 17) or when WT STING was replaced with the STING I200N *golden ticket* mutant that is unable to bind and respond to cyclic dinucleotides (13, 14). Altogether, these results indicate that IFI16-dependent DNA sensing does not induce apoptosis, whereas cGAS-dependent DNA sensing, signaling through the STING/TBK-1/IRF3 axis, does. Furthermore, this function of cGAS requires its enzymatic production of cyclic dinucleotides upon DNA stimulation.

### IFI16 transcriptionally represses HSV-1 gene expression.

**FIG 7:**
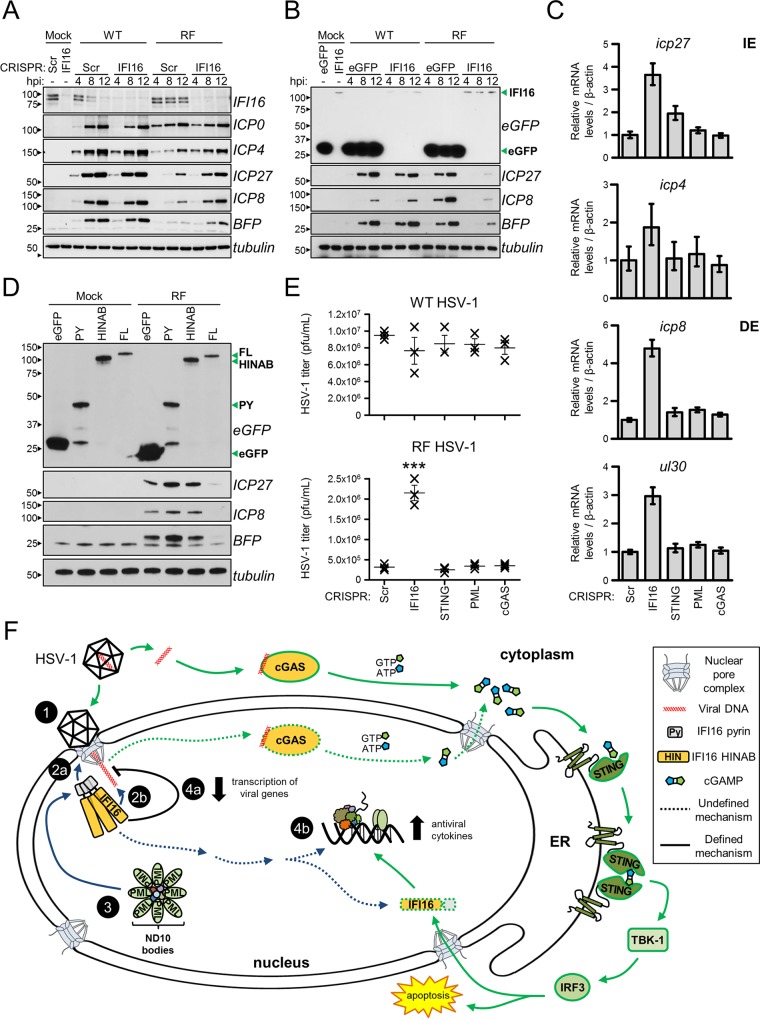
IFI16 suppresses HSV-1 gene expression and viral replication. (A) Western blots of CRISPR-HFFs infected with WT or RF HSV-1 (MOI of 1) for the indicated times. (B) As in panel A, induced Flp-In 293s expressing the indicated constructs (green arrowheads). (C) mRNA levels of immediate early (IE) and delayed early (DE) viral genes in RF HSV-1-infected CRISPR-HFFs (MOI of 1) at 8 hpi. Data normalized to *β-actin*. Values are means ± standard deviations (SD) (*n* = 3). (D) Western blots of RF HSV-1-infected Flp-In 293s expressing indicated IFI16-eGFP fusions (green arrowheads). (E) Progeny WT or RF HSV-1 titers from infected CRISPR-HFFs (MOI of 0.5). Cell-associated and cell-free virus were pooled at 24 hpi, and the titers of the virus on U2OS cells were determined by plaque assay. Values are means ± SEMs (*n* = 3). Values that are significantly different (*P* ≤ 0.001) from the value for the scrambled control by Student’s *t* test are indicated (***). (F) Model for IFI16 and cGAS antiviral functions. Nucleus-replicating DNA viruses, such as HSV-1 and HCMV, deposit their double-stranded DNA (dsDNA) genome into the nucleus (step 1). IFI16 PY domain oligomerizes at the nuclear periphery (step 2a), and HINAB domains bind the viral genome (step 2b). The PY domain interacts with ND10 bodies and their associated protein components (step 3), which may play auxiliary functions in coordinating with full-length (FL) IFI16 to transcriptionally repress viral gene expression (step 4a). IFI16 binding to viral DNA may trigger a noncanonical cytokine signaling pathway that is independent of STING (step 4b). Viral DNA is also sensed by cGAS. The resulting cGAMP production stimulates the STING/TBK-1/IRF3 signaling axis to induce antiviral cytokines and apoptosis. ER, endoplasmic reticulum.

Our prior analyses of endogenous IFI16 interactions revealed transcription factors and chromatin remodeling complexes (24), and the domain-specific studies now link these to the HIN domains. Along these lines, early studies implicated IFI16 in the regulation of genes carried on foreign DNA molecules, viral or recombinant (5, 27, 28). Therefore, to better delineate the antiviral properties of IFI16, we probed its role in regulating HSV-1 gene expression. Viral protein levels were assessed in CRISPR-HFFs infected with either WT or RF derivatives of HSV-1::*bfp* (MOI of 1). Knockout of IFI16 had no observable effect on the levels of either immediate early (IE) (ICP0, ICP27) or delayed early (DE) (ICP8) viral proteins during WT HSV-1 infection (Fig. 7A). The RF mutant is known to be attenuated in viral gene expression. IFI16 knockout rescued this attenuation, as ICP27 and ICP8 levels were higher in IFI16 CRISPR cells than in control cells at 4, 8, and 12 hpi. A rescue effect was also observed for the blue fluorescent protein (BFP) expression cassette present in both recombinant viruses, implicating IFI16 represses expression of genes carried on the viral genome regardless of their origin. In agreement, overexpression of IFI16 in a Flp-In 293 inducible system reduced viral and BFP protein levels during infection with RF, but not WT, HSV-1::*bfp* (Fig. 7B). The regulation at the transcription level was confirmed by reverse transcription-quantitative PCR (RT-qPCR) quantification of viral transcripts. While STING, cGAS, and PML knockouts had little effect on viral transcript abundances, IFI16 knockout elevated the *icp27* (~4-fold) and *icp4* (~2-fold) IE transcripts and the *icp8* (~5-fold) and *ul30* (~3-fold) DE transcripts (Fig. 7C). Furthermore, expression of either IFI16 PY or HINAB domains in Flp-In 293 cells did not suppress viral gene expression, revealing that both structural elements are required for IFI16-mediated viral gene repression (Fig. 7D). To determine whether this transcriptional repression or STING/TBK-1/IRF3-dependent cytokines influenced the production and spread of HSV-1 in culture, we infected CRISPR-HFFs with WT and RF HSV-1 at a low MOI (0.5) and measured viral titers of the progeny. Interestingly, cGAS, STING, and PML knockout had no effect on either WT or RF virus production, whereas IFI16 knockout significantly elevated RF titers. Taken together, these results strongly suggest that IFI16 acts to transcriptionally repress HSV-1 and that ICP0 ubiquitin ligase activity is necessary to eliminate these repressive functions.

## DISCUSSION

IFI16 was demonstrated to bind to DNA of nucleus-replicating herpesviruses (2, 6); however, the full range of its functional outputs is not understood. In this study, we observed the rapid formation of IFI16 subnuclear puncta located exclusively at the nuclear periphery upon infection with herpesviruses HSV-1 and HCMV. This dynamic behavior was maintained during infection with the transcription- and replication-deficient *d109* HSV-1 mutant, suggesting that only the viral nucleocapsid and genome are required to trigger IFI16 peripheral focus formation. The *d109* viral genome has been previously shown to persist within infected nuclei (34), explaining why assembly of peripheral foci continues into later hours of infection. Furthermore, these intranuclear foci were dependent on the MOI and formed instantaneously at sites directly adjacent to where the viral nucleocapsid binds on the outer nuclear membrane. The earliest viral genomes are deposited in the nucleus within 1 h of HSV-1 virion adsorption to the plasma membrane (35). Thus, this early recruitment to the periphery appears to be coordinated with the deposition of the viral genome in both space and time. Considering that the HIN200 domains of IFI16 mediate its binding to viral DNA (6, 36) and its PY domain mediates homotypic oligomerization (37, 38), it is surprising that the PY domain, not the HIN200 domain, is incorporated into these peripheral nuclear puncta. Given our observed interactions between the PY domain and many components of the nuclear pore complex (NPC) during infection, it may be that binding of the viral capsid and subsequent release of the viral DNA induces structural changes at the NPC which actively recruit local IFI16 via PY. This would position free HIN200 domains to bind the incoming viral DNA. Furthermore, our observation that the PY domain interacts with ND10 body proteins PML, Sp110, and SUMO1/2/3 indicates that mobilization of IFI16 may further recruit these cellular intrinsic factors to viral genomes. Indeed, by immunofluorescence microscopy, we observed colocalization of PML with both full-length IFI16 and its PY domain at early stages of infection. Interestingly, three-dimensional (3D) rendering revealed that IFI16 and PML inclusions do not perfectly colocalize and may exist as distinct structures. Given the kinetics of IFI16 redistribution, it is likely that IFI16 foci form first, and subsequently recruit ND10 bodies to the nuclear periphery. Indeed, IFI16 CRISPR-mediated knockout impeded PML recruitment to viral DNA entry points. Our findings that IFI16 expression strongly represses viral IE and DE gene expression and viral replication supports a model in which early recruitment of IFI16 to the periphery serves to silence viral genomes. It is established that ND10 body-localized proteins ATRX and human Daxx (hDaxx) form a chromatin remodeling complex that downregulates HSV-1 gene expression (39). However, we found that PML knockout had little effect on both viral transcription and viral reproduction, suggesting that recruitment of IFI16, not PML bodies, is a critical process in intrinsic antiviral defenses. IFI16-mediated repression of viral gene and transgene expression has been previously reported (5, 40, 41). It is also worth noting that knockout of STING and cGAS had no effect on viral replication in tissue culture, indicating that regulation of viral gene expression functions as a greater barrier to viral replication than the induction of antiviral cytokines.

After peripheral focus formation and disassembly, the cell appears to enter a “relaxation” state marked by diffuse IFI16 nucleoplasmic localization with nucleolar enrichment, as seen in normal, uninfected cells. In the second phase of infection, however, IFI16 is recruited into puncta which are greater in number than in the first phase and distributed throughout the nucleus. We observed that these nuclear foci in the second phase were directly adjacent to centromeres and conferred by the HIN200 domains. As our optogenetic studies suggest the HIN200 domain cannot self-associate, formation of these puncta must be mediated by other cellular factors. Indeed, by proteomics, we observed an enrichment of the HIN200 domain with centromeric protein CENP-B upon HSV-1 infection. Previous studies have demonstrated that HSV-1 ICP0 targets centromeric proteins, including CENP-A, CENP-B, and CENP-C, for degradation and induces a cellular response known as interphase centromere damage response (iCDR) (29, 30). While iCDR is not well understood, it is believed that it functions as a mitotic checkpoint to ensure proper kinetochore formation and eventual chromatid separation in the event of premitotic structural damage to the centromeres. Our data imply that IFI16 is involved in initiating iCDR signaling and expand the role of IFI16 to other cellular stress responses. Further studies are warranted.

In the third phase of HSV-1 infection, IFI16 is almost completely degraded. This degradation is dependent on the PY domain. The E3 ubiquitin ligase ICP0 contributes to proteasome-dependent destabilization of IFI16 but is not sufficient (23, 24). Thus, the responsible E3 ubiquitin ligase remains to be identified. KLHL7 is an adapter protein for Cul3-containing E3 ubiquitin ligase complexes (42), and we found that it uniquely interacts with the PY domain during HSV-1 infection. The PY domain, therefore, may be degraded by Cul3 in response to activities related to ICP0 E3 ubiquitin ligase functions.

In the context of immune signaling, we demonstrate that IFI16 is not an upstream initiator of the STING/TBK-1/IRF3 signaling axis in response to DNA or DNA virus infection, as assessed by CRISPR/Cas9 knockout. However, we also show that IFI16 knockout attenuates cytokine responses to RF HSV-1 infection. Therefore, we propose a model in which IFI16 functions either downstream of this canonical DNA sensing axis or through a noncanonical, nuclear immune signaling pathway. Its redistribution to sites that overlap with ND10 bodies and that are similar in appearance to active transcription sites in response to DNA virus infection (24) suggests that IFI16 may be a transcriptional activator of cytokine expression.

In contrast to IFI16, we show that the DNA sensor cGAS is required for activation of all three integral components of the STING pathway in response to transfected DNA and DNA viruses HSV-1 and HCMV. Furthermore, cGAS-dependent immune signaling additionally leads to the activation of executioner caspase-3 and apoptosis. Induction of apoptotic signaling appears to be dependent on DNA as it additionally requires STING and was elicited in response to both DNA transfection and HSV-1 infection. Furthermore, cGAS production of cyclic dinucleotides and the ability of STING to bind cyclic dinucleotides are required. Previous studies have linked IRF3 activation to Bax-mediated apoptosis during viral infection (43). Thus, we identify cGAS as an inducer of apoptosis in response to foreign DNA through its activation of the STING/TBK-1/IRF3 pathway. The intracellular location of deposition of viral DNA seems to be a major determinant in the activation of either the canonical or noncanonical pathway. During infection, viral nucleocapsids successfully target their cargo DNA to the nucleus, possibly activating either the canonical or noncanonical pathway, dependent on nuclear pools of cGAS or IFI16. As cGAS seems to have both cytoplasmic and nuclear localizations (see Fig. S5F and S5G in the supplemental material), this begs the question whether nuclear populations of cGAS are involved in sensing HSV-1 and HCMV DNA during infection. Alternatively, the nucleocapsids may become destabilized during transit to the nucleus and aberrantly release viral DNA into the cytosol, activating the canonical STING/TBK-1/IRF3-dependent pathway. Indeed, empty capsids depleted of their DNA genomes have been detected in the cytosolic and endosomal compartments upon high-multiplicity infections of HSV-1 (35). This provides a mechanism by which DNA of nucleus-replicating viruses is introduced into the cytoplasmic environment and exposed to cytoplasmic DNA sensors. Future investigations into the nuclear sensing abilities of cGAS are, nevertheless, warranted.

Altogether, we used live-cell imaging to track IFI16 spatial-temporal dynamics in real time. This approach avoids fixation, permeabilization, and immunocytochemical processes that can introduce experimental artifacts, allowing the capture of IFI16 movements in chronological order. We also used CRISPR/Cas9-mediated knockouts in primary human tissue culture to probe the involvement of specific DNA sensing pathway components. Unlike RNA interference technologies (e.g., small interfering RNA [siRNA] and short hairpin RNA [shRNA]), CRISPR/Cas9 confers irreversible knockout of target gene expression in individual cells and avoids antiviral responses triggered by the double-stranded RNA substrates. These important advantages lead to more-reliable and interpretable biological assays and underscore the applicability of CRISPR/Cas9 genetic tools in tissue culture. Finally, optogenetics allows for fine experimental control of specific cellular responses and is, therefore, well suited for interrogating critical immune signaling pathways. To our knowledge, this development and use of optogenetic tools for studying DNA sensing pathways have not been previously reported.

## MATERIALS AND METHODS

Full descriptions of materials and methods are provided in Text S1 in the supplemental material.

### Transfection, transduction, and CRISPR and cell line construction.

For transfection, Lipofectamine 2000 (Life Technologies) was used per the manufacturer’s instructions. Lentiviruses were prepared according to the RNA interference (RNAi) Consortium’s protocols. A total of 5 × 10^5^ HFFs were transduced with lentivirus (2 days) and selected with either puromycin (2 µg/ml) or fluorescence-activated flow cytometry (S3 cell sorter [Bio-Rad]). For CRISPR, candidate 20-bp guide RNA sequences were designed using the CRISPR Design Tool (http://crispr.mit.edu/) and delivered using the LentiCRISPR v2 vector (31) (Addgene plasmid 52961) from Feng Zhang. Flp-In 293 cells were a gift from Loren Runnels.

### Live-cell imaging of IFI16 during viral infection and optogenetics.

IFI16-full-eGFP, IFI16-PY-eGFP, and IFI16-HINAB-eGFP constructs (4) and IFI16-FusionRed (this study) were stably expressed in HFFs via lentivirus transduction. WT and RF HSV-1::*bfp* viruses were generated by Red recombineering-based bacterial artificial chromosome (BAC) mutagenesis as described previously (44). WT HCMV::*gfp* and ΔpUL83 HCMV::*gfp* were provided by Thomas Shenk. *d106*::*gfp* and *d109* HSV-1 mutants were a gift from Neal DeLuca. RF HSV-1 was a gift from Bernard Roizman and Saul Silverstein. HSV-1::*mrfp-vp26* was provided by Lynn Enquist. Cells were imaged on a Nikon TI-E microscope with a Spinning Disc (Orca Flash charge-coupled-device [CCD] camera [Hamatsu]) and Perfect Focus system using either a 100× or 60× objective. Maximum signal-to-noise pixel intensity ratios were calculated ImageJ (NIH). Statistical significance of puncta counts was assessed by one-way analysis of variance (ANOVA) with Bonferroni’s test (GraphPad Prism). FusionRed-Cry2^olig^-IFI16 and its derivatives were stimulated with 100-ms pulses of 488-nm laser every minute and imaged on a Nikon Eclipse Ti microscope. For all live-cell imaging experiments, a minimum of 10 fields were visualized, containing a total of 20 to 100 cells per experiment.

### RNA isolation and quantitative RT-PCR.

Total cellular RNA was extracted from 3 × 10^5^ HFFs using a RNeasy minikit (Qiagen). cDNA was reverse transcribed from 1 µg RNA using RETROscript reverse transcription kit (Life Technologies), and target transcripts were quantified by qPCR using the SYBR green PCR master mix (Life Technologies). Transcript levels were normalized to *β-actin* or *gapdh* levels and quantified using the ΔΔ*C_T_* method.

### Identification of PY- and HIN-protein interactions by IP coupled to mass spectrometry.

HFF cells stably expressing either FL IFI16-eGFP, PY-eGFP, HIN-eGFP, or eGFP alone were infected with RF HSV-1 (MOI of 10). Cells were harvested 6 hpi, and immunoaffinity isolations with a GFP antibody prepared in-house were conducted as described previously (24). Eluted proteins were digested with trypsin (Promega) and analyzed by nano-liquid chromatography coupled to tandem mass spectrometry (nano-LC-MS/MS) with an ESI-LTQ Orbitrap Velos (Thermo Scientific). Raw MS/MS spectra were searched and extracted using Proteome Discoverer (v. 1.4), and assessed through SEQUEST HT (v. 1.4). Specificity filtering on weighted spectral counts was conducted using the SAINT (Significance Analysis of INTeractions) algorithm, and a value of 0.96 was used as a specificity threshold (see Table S1 in the supplemental material). Spectral counts were normalized by the length of the IFI16 bait (PY or HINAB) and log_2_ fold enrichment was assessed as PY/HIN. The specific proteins were submitted to the STRING database (45), categorized by Gene Ontology, and visualized in Cytoscape (v. 3.4.0).

## SUPPLEMENTAL MATERIAL

Text S1  Supplemental experimental procedures Download Text S1, DOCX file, 0.04 MB

Figure S1  IFI16 localization is static in uninfected HFFs and asymmetric in the nucleus during HSV-1 infection. (A) HFFs infected with RF HSV-1 (MOI of 0.1) at 24 hpi. Representative cell shown (white arrow) is at the edge of a plaque. Bar, 10 µm. (B) HFFs expressing IFI16-mEGFP (top) or IFI16-FusionRed (bottom) were mock infected and imaged by live-cell confocal fluorescence microscopy. Download Figure S1, TIF file, 0.5 MB

Figure S2  HSV-1 DNA and ICP0 activity are sufficient for IFI16 localization to sites of HSV-1 DNA deposition. HFFs expressing IFI16-FusionRed were infected with HSV-1 *d106*::*gfp* (MOI of 10) and monitored by live-cell fluorescence confocal microscopy. Dynamic puncta are indicated (white arrows). Bar, 5 µm. Download Figure S2, TIF file, 0.3 MB

Figure S3  In the absence of endogenous IFI16, IFI16 PY and HIN domains display distinct behaviors during HSV-1 infection. (A) Immunofluorescence images of Flp-In 293 cells expressing either eGFP or eGFP-tagged IFI16 domains (PY, HIN, FL). Cells were infected with RF HSV-1 at an MOI of 10 and imaged at 6 hpi. Colocalization is indicated (white arrows). Bar, 10 µm. (B and C) Western blots of HFF cells (B) or Flp-In 293 cells (C) as described above for panel A. Cells were mock infected or infected with either WT HSV-1 or RF HSV-1 at an MOI of 10 at 6 hpi. (D) Lower exposures of Western blots as described above for panel C. (E) HFFs expressing IFI16-eGFP were infected with WT HSV-1 (MOI of 10) and monitored by live-cell fluorescence confocal microscopy. Colocalization between IFI16 and centromeres are indicated (white arrows). Download Figure S3, TIF file, 0.7 MB

Figure S4  SAINT specificity scores and immunoaffinity isolation of IFI16 with cGAS. (A and B) Binned distribution of prey protein average pSAINT scores (*n* = 2) for IFI16-PY (A) and -HIN (B) isolations. (C) eGFP immunoaffinity isolations from HEK293T cells cotransfected with the indicated IFI16-eGFP fusion (black arrows) and FLAG-cGAS. Download Figure S4, TIF file, 0.1 MB

Figure S5  CRISPR/Cas9-mediated knockout in primary human foreskin fibroblasts. (A to D) Western blots of CRISPR-HFFs expressing one of three candidate *ifi16*, *sting*, *pml*, and *cgas*-specific guide RNAs. Constructs with red asterisks were used for all subsequent experiments. (E) Immunofluorescence microscopy of ICP4 and PML in CRISPR-HFFs (sgcGAS and sgSTING) upon RF HSV-1 infection (MOI of 0.1) at 24 hpi. A representative cell shown is at the edge of a plaque. Bar, 10 µm. (F) As in panel E, immunofluorescence microscopy of ICP4 and cGAS in CRISPR-HFFs (sgIFI16, sgPML, and sgSTING versus sgScr). (G) Localization of IFI16-mCherry and cGAS-GFP in HFF cell lines, stably expressing the constructs. Download Figure S5, TIF file, 0.7 MB

Table S1  Spectral counts, SAINT specificity pScores, and Gene Ontology (GO) annotations for each biological replicate from HFFs infected with RF HSV-1 expressing Py-GFP, HIN-GFP, and GFP Table S1, XLSX file, 0.2 MB

Movie S1  Dynamic IFI16 behavior during HSV-1 and HCMV infection. Download Movie S1, MOV file, 4.2 MB

Movie S2  Dynamic IFI16 behavior during HSV-1 *d106*, *d109*, and *mRFP-vp26* infection. Download Movie S2, MOV file, 2.9 MB

Movie S3  IFI16-PY and -HIN domain behaviors during HSV-1 infection and optogenic manipulation. Download Movie S3, MOV file, 3.5 MB
