# Supplementary material for: Viral DNA Sensors IFI16 and Cyclic GMP-AMP Synthase Possess Distinct Functions in Regulating Viral Gene Expression, Immune Defenses, and Apoptotic Responses during Herpesvirus Infection
Source: mBio. 2016 Nov 15;7(6):e01553-16. doi: 10.1128/mBio.01553-16 (PMC5111403; doi:10.1128/mBio.01553-16)
Supplement: Text S1 — Supplemental experimental procedures Download [file mbo006163072s1.docx]

**Supplementary Experimental Procedures**

**Plasmids and chemical reagents**

To generate pHR-MCS, a multiple cloning site was ligated into pHR, between *BamHI* and *NotI* restriction sites. pHR lentiviral vector backbone was a gift from Dr. Jared Toettcher of Princeton University (Princeton, NJ, USA). IFI16-full-EGFP, IFI16-PY-EGFP and IFI16-HINAB-EGFP constructs have been previously described ([Li et al., 2013](#_ENREF_39)). Each of these IFI16-EGFP fusions were cloned into pHR-MCS using *SpeI* and *NotI* restriction sites. The FusionRed open reading frame ([Shemiakina et al., 2012](#_ENREF_54)) (available from Evrogen) was synthesized as a geneBlock (Integrated DNA Technologies) and cloned into pcDNA3.1 using *BamHI* and *XbaI* restriction sites. IFI16-FusionRed was generated using overlap extension PCR and ligated into pHR-MCS using *SpeI* and *NotI* restriction sites. LentiCRISPRv2 vector was a gift from Feng Zhang (Addgene plasmid # 52961) ([Sanjana et al., 2014](#_ENREF_52)). All sgRNAs were cloned into lentiCRISPRv2 using the *BsmBI* restriction site. LentiORF pLEX-MCS was purchased from Open Biosystems. cDNA of FLAG-hcGAS and HA-hSTING (THP-1) were gifts from Dr. Russel Vance of University of California Berkley (Berkley, CA, USA) ([Diner et al., 2013](#_ENREF_11)). The I200N mutation of hSTING (THP-1) analogous to that of the *golden* *ticket* mutation in mSTING ([Sauer et al., 2011](#_ENREF_53)) was generated by a site-directed mutagenesis method, as described ([Liu and Naismith, 2008](#_ENREF_42)). The GS212-213AA mutation of hcGAS analogous to that of the GS198-199AA mutation of mcGAS ([Sun et al., 2013](#_ENREF_59)) was generated in the same manner. hcGAS-EGFP was cloned by overlap-extension PCR and ligated into pcDNA3.1 using *KpnI* and *XhoI* restriction sites. cDNA of PML isoform I was kindly provided by Dr. Peter Hemmerich of Leibniz Institute on Aging - Fritz Lipmann Institute (Jena, Germany). A FLAG tag was incorporated at the 5’ end of the PML-I open reading frame and cloned into pcDNA3.1 using *BamHI* and *NotI* sites.

**Tissue culture and cell line construction**

HEK293T, HEK293 Flp-In T-Rex, Vero, primary human foreskin fibroblasts (HFFs), and U2OS cells were all cultured in Dulbecco's Modified Eagle medium (Life Technologies) supplemented with 50 I.U./mL penicillin, 50 µg/mL streptomycin, and 20% fetal bovine serum (Atlanta Biologicals) at 37°C in 5% CO_2_. HEK293 Flp-In T-Rex cells were generously provided by Dr. Loren Runnels of University of Medicine and Dentistry of New Jersey/Robert Wood Johnson Medical School (New Brunswick, NJ, USA). HFFs were generously provided by Dr. Hillary Coller of University of California – Los Angeles (Los Angeles, CA, USA). For UV treatment experiments, cells were irradiated with 10 or 50 Joules/m^2^ using a Stratagene Stratalinker 2400 UV Crosslinker.

Flp-In T-Rex HEK293 cell lines inducibly expressing IFI16-EGFP domain fusions were constructed as follows: pcDNA5/FTR/TO (Invitrogen) plasmid carrying indicated gene constructs and pOG44 Flp recombinase vector (Invitrogen) were co-transfected for 48 hours. After this time, media was supplemented with 150 μg/mL Hygromycin B (Life Technologies) to select for positive transfectants. After one week of selection, resistant colonies were picked and expanded. 24 hours prior to assaying, EGFP fusion protein expression was induced with 1 μg/mL tetracycline (Sigma-Aldrich) for all inducible cell lines. To construct the HEK293T-STING cell line, HEK293T cells were transduced with lentivirus derived from LentiORF pLEX-MCS carrying the STING (THP-1) open reading frame and selected with 5 µg/mL puromycin for two weeks. Puromycin-resistant colonies were pooled and expanded. For constructing all HFF cell lines expressing either CRISPR/Cas9/sgRNA cassettes or fluorescent fusion proteins, HFFs were transduced with the respective lentiviruses for three days. Positive transductants were then selected by either 2 µg/mL puromycin for one week (for CRISPR/Cas9) or fluorescence-activated flow cytometry (S3 Cell Sorter (BioRad)) (for fluorescent fusion proteins).

**Viruses and BAC mutagenesis**

All LentiORF pLEX-MCS-, LentiCRISPRv2-, and pHR-based lentiviruses were packaged and harvested from HEK293T cells in the same manner. Briefly, lentiviral packaging vectors psPAX2 and pMD2.G (VSV-G) were co-tansfected with one of the aforementioned lentiviral transfer vectors in a ratio of 2.25:1.5:1.5 (psPAX2:pMD2.G:transfer vector) using X-tremeGene HP transfection reagent at a ratio of 1:2 (DNA:X-tremeGene HP). Lentivirus was collected at 48, 72, and 96 hours post-transfection in 30% FBS DMEM supplemented with 25mM HEPES, pH 7.4 and filtered through a 0.45 µm membrane. Lentivirus-containing supernatants were subjected to ultracentrifugation (25,000 rpm, 2 hours, 4°C with SW28 swinging bucket rotor (Beckman Coulter)). Viral pellets were solubilized in PBS overnight at 4°C, aliquoted, and frozen at -80^o^C until use.

A bacterial artificial chromosome carrying the full HSV-1 (17+ strain) genome carried by *E. coli* strain GS1783 with inducible Red recombination system and I-SceI expression were a gift from Dr. Beatte Sodeik of Hannover Medical School (Hannover, Germany). Wild-type HSV-1 was reconstituted via electroporation of pBAC-HSV-1 into Vero cells. Both wild-type HSV-1 and the *ICP0-RF* viruses were expanded and titered in U2OS cells. The *d106* virus (ICP0^+^ ICP4^-^ ICP27^-^) was expanded and titered in ICP4- and ICP27-complementing E11 (Vero) cells, as described ([Samaniego et al., 1998](#_ENREF_50)). To propagate virus, the indicated cell cultures were infected at low multiplicities of infection (MOI = 0.001) and infection proceeded until 100% cytopathic effect was observed (3-4 days). Both culture supernatant and cells were collected and buffered with MNT buffer (200 mM MES, 30mM Tris-HCl, 100 mM NaCl, pH 7.4). Supernatants were subjected to ultracentrifugation (20,000 rpm, 2 hours, 4°C with SW28 swinging bucket rotor (Beckman Coulter)) to concentrate virus. Cell-associated virus was collected by sonication and pooled with pelleted cell-free virus. All virus stock titers were determined by plaque assay on their respective propagator cell type. For all infections, viral stocks were diluted in 2% (v/v) FBS-containing DMEM to the indicated MOI and added to cell monolayers for 1 h at 37^o^C with intermittent rocking. After viral adsorption, cells were washed once with PBS, replenished with DMEM containing 10% (v/v) FBS, and incubated at 37^o^C for the indicated period of time. The *ICP0-RF* mutant was a gift from Dr. Bernard Roizman of University of Chicago (Chicago, IL, USA) and Dr. Saul Silverstein of Columbia University (New York, NY, USA). Both the *d106* mutant and complementing E11 cells were gifts from Dr. Neal DeLuca of University of Pittsburgh (Pittsburgh, PA, USA). ΔpUL83-GFP human cytomegalovirus (HCMV) mutant was kindly provided by Dr. Wade Bresnahan of University of Minnesota (Minneapolis, MN, USA).

HSV-1::*tagBFP2* was generated by Red recombination-based BAC mutagenesis as described ([Tischer et al., 2010](#_ENREF_63)). Briefly, the *kanR* gene expression cassette of pEGFP-N1 flanked by a 50 base pair internal TagBFP2 repeat and the 18 base pair *I-SceI* restricton site was inserted into the TagBFP-NLS expression cassette using the *BsrGI* restriction site. A 50 base pair sequence homologous to the intergenic region between HSV-1 UL26 and UL27 genes was added to each side of the *tagBFP2-NLS*/*kanR* expression cassette by PCR and the amplicon was transformed into *E. coli* strain GS1783 carrying pBAC-HSV-1 (17+). Transformants were selected by kanamycin (amplicon) and chloramphenicol (BAC) and positive co-integrates were confirmed by PCR. Resolution of the *kanR* cassette is achieved by sequential induction of I-SceI expression via 1µg/mL L-arabinose and Red recombination system expression via brief incubation at 42°C. Successfully resolved colonies were identified based on sensitivity to kanamycin by replica plating and confirmed by PCR. HSV-1::*tagBFP2* virus was reconstituted by electroporation of the resulting pBAC-HSV-1::*ul26­-tagBFP2-ul27* into Vero cells and virus was expanded and titered on U2OS cells, as above.

**Antibodies and Reagents**

The following antibodies were used Western blotting and immunofluorescence microscopy: α-IFI16 (ab50004 and ab55328; Abcam), α-ICP0 (H1A027-100; Virusys Corporation), α-ICP27 (vP-20) (sc-17544 ; Santa Cruz Biotechnology), α-ICP8 (sc-53329; Santa Cruz Biotechnology), α-tubulin (T6199; Sigma-Aldrich), α-PARP (#9542; Cell Signaling Technology), α-caspase-1 p20 (D7F10) (#3866; Cell Signaling Technology), α-caspase-1 p10 (C-20) (sc-515; Santa Cruz Biotechnology), α-cGAS (MB21D1) (HPA031700; Sigma), α-GFP (11814460001; Roche), α-TBK-1 (#3013; Cell Signal Technology), α-Phosph.-TBK-1 (Ser172) (#5483, Cell Signal Technology), α-STING (TMEM-173) ( ab92605; Abcam), α-IRF-3 (FL-425) (sc-9082; Santa Cruz Biotechnology), α-PML (sc-9862; Santa Cruz Biotechnology), α-centromere (#15-234-0001, Antibodies Inc.). For immunoaffinity purification, α-FLAG M2 (F3165; Sigma) and α-GFP (in-house) were used. For generating lentiviruses in HEK293T packaging cells, X-tremeGENE HP transfection reagent (Roche) was used according to the manufacturer’s instructions. For all other transfection experiments, Lipofectamine 2000 (Life Technologies) was used per the manufacturer’s instructions.

**SDS-PAGE and Native PAGE**

For reducing SDS-PAGE, cells were lysed in 1x Laemlli buffer (62.5 mM Tris-HCL, pH 6.8, 2% SDS (w/v), 10% glycerol (v/v), 0.02% bromophenol blue (w/v), 100 mM DTT) and boiled at 95^o^C for 10 minutes. For non-reducing SDS-PAGE, cells were lysed in RIPA buffer (50 mM Tris, pH 7.5, 150 mM NaCl, 1% NP-40, 0.1% SDS, 0.5% sodium deoxycholate (DOC), 1 mM EDTA, 1/100 PIC) and incubated on ice for 30 minutes with intermittent vortexing. Lysates were clarified with centrifugation at 10,000x*g* for 10 min at 4^o^C. Supernatants were extracted and further solubilized with Laemlli loading buffer (as above) without DTT. Samples were then heated at 70^o^C for 20 minutes, bath sonicated, and centrifuged at 15,000 rpm for 10 minute to pellet cell debris prior to electrophoresis by SDS-PAGE.

For native-PAGE, cells were lysed with native lysis buffer (50 mM Tris-HCl, pH 7.5, 150 mM NaCl, 1 mM EDTA, 0.7% NP-40, 10% glycerol, and 1/100 PIC) and incubated on ice for 30 minutes with intermittent vortexing. Lysates were clarified with centrifugation at 10,000x*g* for 10 min at 4^o^C. Cleared supernatants were extracted and supplemented with native-PAGE sample buffer (62.5 mM Tris-HCl, pH 6.8, 10% glycerol, 1% sodium DOC). PAGE gels were made in the usual fashion without SDS. Native-PAGE gels were pre-run at 40 mA for 30 min in 25 mM Tris, 192 mM Glycine, pH 8.4 with and without 1% DOC in the cathode and anode chambers, respectively, in order to equilibrate with DOC. Samples were then electrophoresed for 60 min at 20 mA at 4^o^C. For all PAGE, proteins were electroblotted onto PVDF membrane before proceeding with standard Western blotting practices.

**RNA isolation and quantitative RT-PCR**

The RNeasy Mini kit (Qiagen) was used to column purify total cellular RNA per the manufacturer’s instructions. DNA contaminants were digested with DNAseI (Invitrogen) for 15 minutes at room temperature. RNA (1 µg) was reverse transcribed using oligo-dT primers and RETROscript Reverse Transcription kit (Life Technologies). Gene-specific primers were used to quantify the resulting first-strand cDNA by qPCR using the SYBR green PCR master mix (Life Technologies) on an AB7900HT thermocycler (Applied Biosystems). The ∆∆CT method was applied to determine relative mRNA quantities, normalizing mRNA across samples to *β-actin* or *gapdh* transcript levels.

**Fluorescence Microscopy and Live Cell Imaging**

For both fixed and live cell imaging experiments, cells were seeded on 8-well Nunc™ Lab-Tek™ II Chambered Coverglass system (ThermoScientific) and given 24 hours to attach prior to experimental manipulation. For fixing and immunocytochemistry, cells were fixed in 2% (v/v) paraformaldehyde in phosphate-buffered saline (PBS) for 15 min, permeabilized with 0.1% (v/v) Triton-X in PBS for 15 min, and blocked with 2% (w/v) BSA PBS-T (0.2% (v/v) Tween-20 in PBS). Fixed and permeabilized cells were probed first with primary antibody diluted in 2% BSA PBS-T for 1 hour, and then probed with secondary antibody conjugated with Alexa Fluorophores (Life Technologies) also in 2% BSA PBS-T. Nuclei were additionally stained using 1 µg/ml DAP for 10 min. All steps were carried out at room temperature. Cells were imaged on a Nikon TI-E with Spinning Disc (Orca Flash CCD camera (Hamatsu)) and Perfect Focus System. For live cell imaging experiments, cells were infected as described above and were maintained at 37^o^C and 5% CO_2_ using an environmental control chamber. To avoid photobleaching during long-term imaging of live cells, lasers were not used at power levels greater than 20% and exposures were kept below 100 ms. For all experiments, cells were imaged with either 63x or 100x oil immersion objectives. Data collection and processing was performed using NIS-Elements software v.4.10.01 (Nikon).

**Immunoaffinity purifications of IFI16, PML, and cGAS**

Cells were washed once with PBS, trypsinized, and collected by scraping. After pelleting by brief centrifugation (300x*g*), cells were lysed with lysis buffer (20 mM K-HEPES, pH 7.4, 0.11 M KOAc, 2 MM MgCl_2_, 0.1% Tween-20 (v/v), 1 µM ZnCl_2_, 1 µM CaCl_2_, 0.6% Triton X-100, 200 mM NaCl, 100 U/mL benzonase, and 1/100 PIC) on ice, for 30 min, with intermittent vortexing. Cell lysates were clarified by centrifugation at 10,000x*g* for 10 minutes at 4^o^C and immunoprecipitated with 1 mg of Dynabeads Magnetic Beads (ThermoScientific) conjugated with either α-FLAG or α-GFP antibody (5µg antibody/mg magnetic beads) for 1 hour at 4^o^C. Antibody-conjugated magnetic beads were prepared as previously described ([Diner et al., 2015](#_ENREF_9); [Diner, 2015](#_ENREF_10); [Luo et al., 2010](#_ENREF_44)). Beads were then washed six times with lysis buffer and immunocomplexes were eluted by boiling in 1x Laemlli buffer (62.5 mM Tris-HCL, pH 6.8, 2% SDS (w/v), 10% glycerol (v/v), 0.02% bromophenol blue (w/v)). Samples were reduced in 100mM DTT at 70^o^C for 10 min. Co-isolated proteins were visualized by Western blotting.

**Isolation of PY- and HIN- protein complexes and mass spectrometry**

HFF cells stably expressing either FL IFI16-eGFP, PY-eGFP, HIN-eGFP, or eGFP alone were infected with RF HSV-1 (MOI 10). Cells (5 x 15cm plates) were harvested after 6 hpi and snap frozen in 20mM Na-HEPES, 1.2% polyvinylpyrrolidone (w/v), pH 7.4. Frozen cell samples were ground in a Retch MM301 Mixer Mill for 10 rounds at 30.0 Hz for 1.5min each. PY-eGFP and HIN-eGFP samples were conducted in biological triplicate, whereas eGFP control samples were conducted in biological duplicate, and the confirmatory FL IFI16-eGFP sample was conducted in one biological replicate. Each ground cell powder replicate was resuspended in 5 ml of lysis buffer (20 mM K-HEPES, pH 7.4, 0.11 M KOAc, 2 mM MgCl_2_, 0.1% Tween 20 (v/v), 1 M ZnCl_2_, 1 M CaCl_2_, 0.6% Triton X-100, 200 mM NaCl, 100 U/ml Universal Nuclease for cell lysis (Pierce), 1⁄100 protease inhibitor mixture (Sigma), 1⁄100 phosphate inhibitor mixtures 2 and 3 (Sigma)). Cell lysates were incubated at room temperature for 10 min to activate DNase and RNase activity, and subjected to homogenization by for 30 s at 20,000 rpm using a PT 10-35 GT Polytron (Kinematica, Bohemia, NY). Lysates were clarified by centrifugation at 8,000 ×g for 10 min at 4^o^C. Per immunoaffinity purification, 5 mg of magnetic M-270 epoxy beads (Life Technologies) were conjugated with 5 µg/mg of in-house GFP antibody. The conjugated magnetic beads were added to the clarified lysate and incubated at 4^o^C for 1 h. The isolated proteins were reduced with tris(2-carboxyethyl)phosphine and alkylated with chloroacetamide, followed by separation on a NuPAGE gel (Invitrogen) and in-gel digestion with trypsin (Promega). Peptides were analyzed by nano-LC-MS/MS with an ESI-LTQ Orbitrap Velos (Thermo Scientific). Data-dependent acquisition mode was used and peptide precursors were subjected to MS/MS fragmentation by collision induced dissociation of the top 15 most abundant precursor ions.

Raw MS/MS spectra was searched and extracted using Proteome Discovered (v. 1.4), and assessed through SEQUEST HT (v. 1.4) against a database containing both human and herpesvirus sequences (Swiss-Prot). A peptide false discovery rate of 1% and a protein minimum of 99% were set in Scaffold (v. 4.0). Proteins were grouped using protein cluster analysis and spectral counts derived from protein clusters were weighted to represent each protein. Specificity filtering was conducted on weighted spectral counts using the Significance Analysis of INTeractions (SAINT) algorithm and a value of 0.96 was used as a specificity threshold (Table S1). The top two highest spectral counts and SAINT specificity scores for identified proteins in each of three biological replicates for PY-eGFP and HIN-eGFP IP-MS samples were averaged. Proteins passing the 0.96 SAINT cut-off score in either the PY or HIN IPs were further analyzed. To compare PY-interacting proteins with HINAB-interacting proteins, each averaged weight spectral count was normalized by dividing the weighted spectral counts by the amino acid length of the IFI16 bait (PY or HINAB). Log-2 fold-enrichment of spectral counts were assessed as PY/HIN. The identified proteins were submitted to the STRING database to import interaction data, categorized by GO classifications, and visualized in Cytoscape (v. 3.4.0).

**Supplementary Figure Legends**

**Figure S1. IFI16 localization is static in uninfected HFFs and asymmetric in the nucleus during HSV-1 infection.**

(A) HFFs infected with RF HSV-1 (MOI 0.1) at 24 hpi. Representative cell shown (white arrow) is at the edge of a plaque. Scale bar, 10 µm.

(B) HFFs expressing IFI16-mEGFP (top) or IFI16-FusionRed (bottom) were mock-infected and imaged by live cell confocal fluorescence microscopy.

**Figure S2. HSV-1 DNA and ICP0 activity are sufficient for IFI16 localization to sites of HSV-1 DNA deposition.**

HFFs expressing IFI16-FusionRed were infected with HSV-1 *d106*::*gfp* (MOI 10) and monitored by live cell fluorescence confocal microscopy. Dynamic puncta are indicated (white arrows). Scale bar, 5 µm.

**Figure S3. In the absence of endogenous IFI16, IFI16 PY and HIN domains display distinct behaviors during HSV-1 infection.**

(A) Immunofluoresence images of Flp-In 293 cells expressing either eGFP, or eGFP-tagged IFI16 domains (PY, HIN, FL). Cells were infected with RF HSV-1 at MOI 10 and imaged at 6 hpi. Co-localization is indicated (white arrows). Scale bar, 10 µm.

(B,C) Western blots of HFF cells (B), or Flp-In 293 cells (C), as in (A). Cells were mock-infected, or infected with either WT HSV-1 or RF HSV-1 at MOI 10, 6 hpi.

(D) Lower exposures of Western blots as in (C).

(E) HFFs expressing IFI16-eGFP were infected with WT HSV-1 (MOI 10) and monitored by live cell fluorescence confocal microscopy. Co-localization between IFI16 and centromeres are indicated (white arrows).

**Figure S4. SAINT specificity scores and immunoaffinity isolation of IFI16 with cGAS.**

(A,B) Binned distribution of prey protein average pSAINT scores (n = 2) for IFI16-PY (A) and –HIN (B) isolations.

(C) eGFP immunoaffinity isolations from HEK293T cells co-transfected with the indicated IFI16-eGFP fusion (black arrows) and FLAG-cGAS.

**Figure S5. CRISPR/Cas9-mediated knockout in primary human foreskin fibroblasts**

(A,B,C,D) Western blots of CRISPR-HFFs expressing one of three candidate *ifi16*, *sting*, *pml*, and *cgas*-specific guide RNAs. Constructs with red asterisks were used for all subsequent experiments.

(E) Immunofluorescence microscopy of ICP4 and PML in CRISPR-HFFs (sgcGAS and sgSTING) upon RF HSV-1 infection (MOI 0.1) at 24 hpi. Representative cell shown is at the edge of a plaque. Scale bar, 10 µm.

(F) As in (E), of ICP4 and cGAS in CRISPR-HFFs (sgIFI16, sgPML, sgSTING, versus sgScr).

(G) Localization of IFI16-mCherry and cGAS-GFP in HFF cell lines, stably expressing the constructs.

**Table S1.** Spectral counts, SAINT specificity pScores, and GO annotations for each biological replicate from Py-GFP, HIN-GFP, and GFP expressing HFFs infected with RF HSV-1.

**Movie S1. Dynamic IFI16 behavior during HSV-1 and HCMV infection**

**Movie S2. Dynamic IFI16 behavior during HSV-1 *d106, d109*, and *mRFP-vp26* infection**

**Movie S3. IFI16-PY and –HIN domain behaviors during HSV-1 infection and optogenic manipulation**
