# Supplementary figures and images for: Viral DNA Sensors IFI16 and Cyclic GMP-AMP Synthase Possess Distinct Functions in Regulating Viral Gene Expression, Immune Defenses, and Apoptotic Responses during Herpesvirus Infection
Source: mBio. 2016 Nov 15;7(6):e01553-16. doi: 10.1128/mBio.01553-16 (PMC5111403; doi:10.1128/mBio.01553-16)

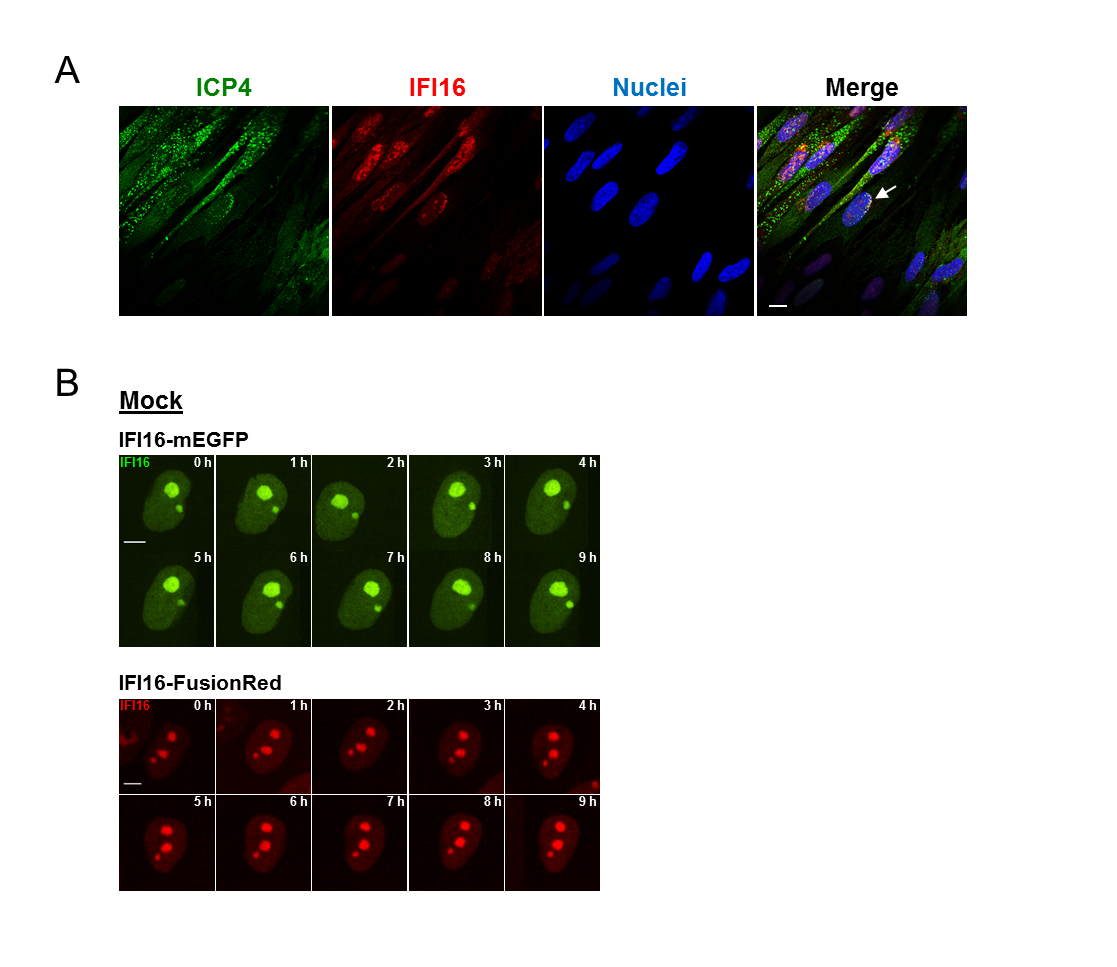

Supplement: Figure S1 — IFI16 localization is static in uninfected HFFs and asymmetric in the nucleus during HSV-1 infection. (A) HFFs infected with RF HSV-1 (MOI of 0.1) at 24 hpi. Representative cell shown (white arrow) is at the edge of a plaque. Bar, 10 µm. (B) HFFs expressing IFI16-mEGFP (top) or IFI16-FusionRed (bottom) were mock infected and imaged by live-cell confocal fluorescence microscopy. Download [file mbo006163072sf1.tif]

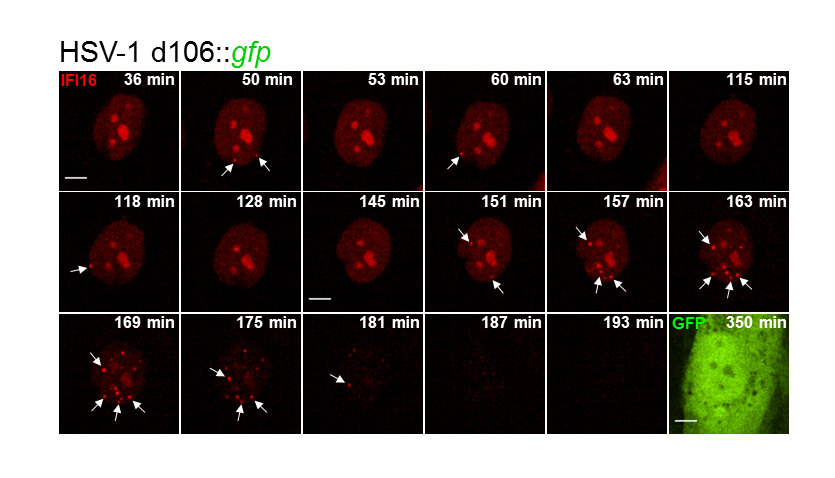

Supplement: Figure S2 — HSV-1 DNA and ICP0 activity are sufficient for IFI16 localization to sites of HSV-1 DNA deposition. HFFs expressing IFI16-FusionRed were infected with HSV-1 d106::gfp (MOI of 10) and monitored by live-cell fluorescence confocal microscopy. Dynamic puncta are indicated (white arrows). Bar, 5 µm. Download [file mbo006163072sf2.tif]

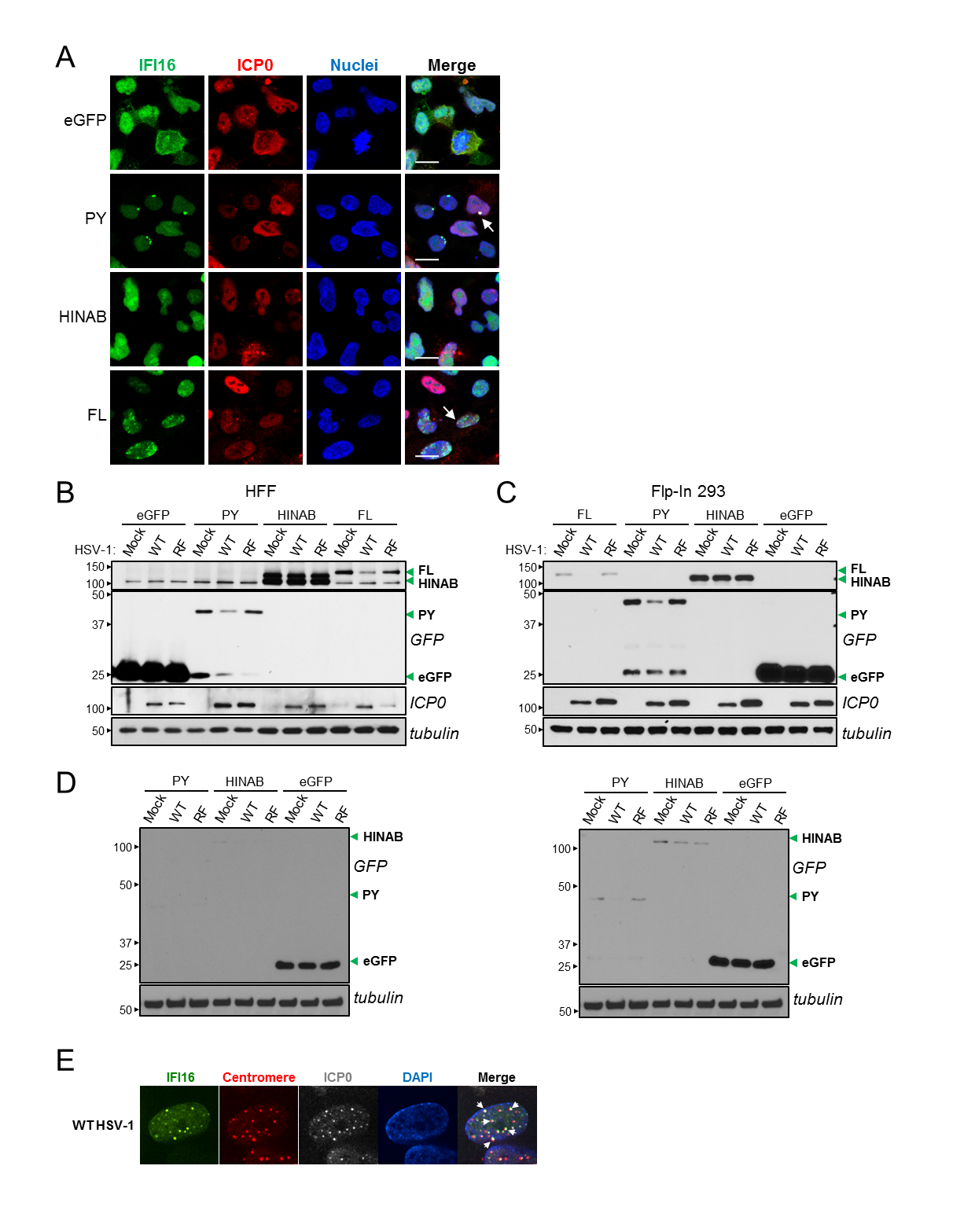

Supplement: Figure S3 — In the absence of endogenous IFI16, IFI16 PY and HIN domains display distinct behaviors during HSV-1 infection. (A) Immunofluorescence images of Flp-In 293 cells expressing either eGFP or eGFP-tagged IFI16 domains (PY, HIN, FL). Cells were infected with RF HSV-1 at an MOI of 10 and imaged at 6 hpi. Colocalization is indicated (white arrows). Bar, 10 µm. (B and C) Western blots of HFF cells (B) or Flp-In 293 cells (C) as described above for panel A. Cells were mock infected or infected with either WT HSV-1 or RF HSV-1 at an MOI of 10 at 6 hpi. (D) Lower exposures of Western blots as described above for panel C. (E) HFFs expressing IFI16-eGFP were infected with WT HSV-1 (MOI of 10) and monitored by live-cell fluorescence confocal microscopy. Colocalization between IFI16 and centromeres are indicated (white arrows). Download [file mbo006163072sf3.tif]

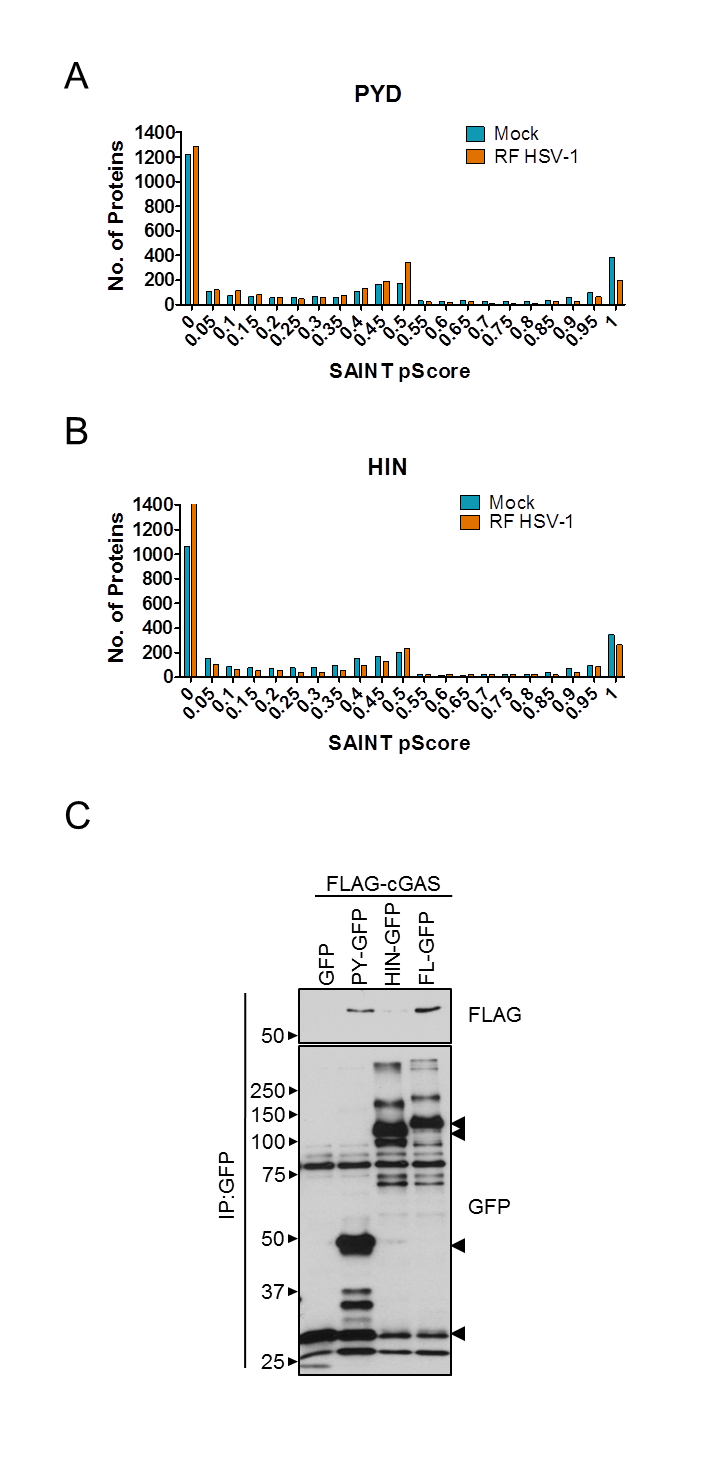

Supplement: Figure S4 — SAINT specificity scores and immunoaffinity isolation of IFI16 with cGAS. (A and B) Binned distribution of prey protein average pSAINT scores (n = 2) for IFI16-PY (A) and -HIN (B) isolations. (C) eGFP immunoaffinity isolations from HEK293T cells cotransfected with the indicated IFI16-eGFP fusion (black arrows) and FLAG-cGAS. Download [file mbo006163072sf4.tif]

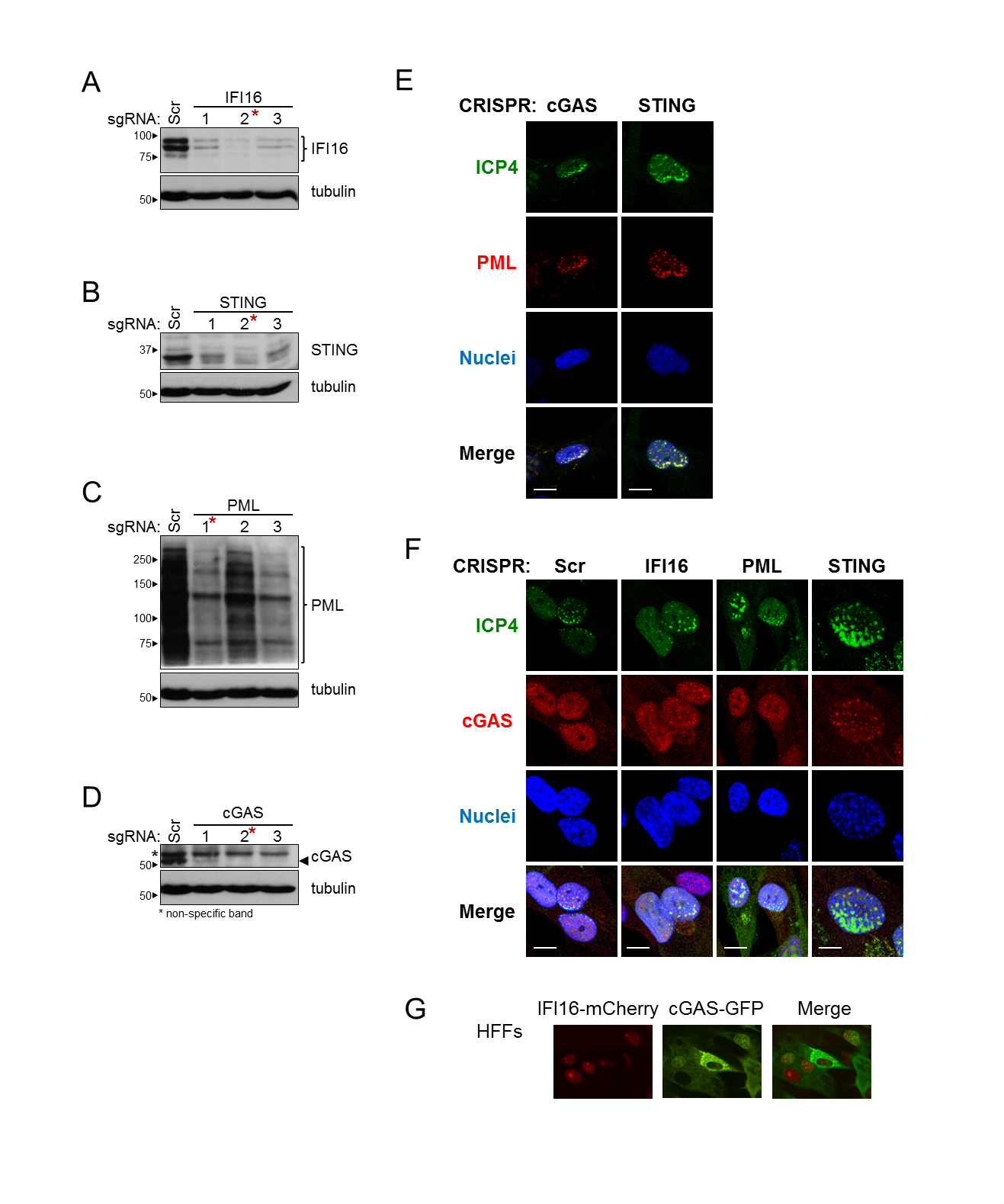

Supplement: Figure S5 — CRISPR/Cas9-mediated knockout in primary human foreskin fibroblasts. (A to D) Western blots of CRISPR-HFFs expressing one of three candidate ifi16, sting, pml, and cgas-specific guide RNAs. Constructs with red asterisks were used for all subsequent experiments. (E) Immunofluorescence microscopy of ICP4 and PML in CRISPR-HFFs (sgcGAS and sgSTING) upon RF HSV-1 infection (MOI of 0.1) at 24 hpi. A representative cell shown is at the edge of a plaque. Bar, 10 µm. (F) As in panel E, immunofluorescence microscopy of ICP4 and cGAS in CRISPR-HFFs (sgIFI16, sgPML, and sgSTING versus sgScr). (G) Localization of IFI16-mCherry and cGAS-GFP in HFF cell lines, stably expressing the constructs. Download [file mbo006163072sf5.tif]
